# Analysis of the HD-Zip I transcription factor family in *Salvia miltiorrhiza* and functional research of *SmHD-Zip12* in tanshinone synthesis

**DOI:** 10.7717/peerj.15510

**Published:** 2023-06-27

**Authors:** Yanhong Bai, Ying Zhou, Qiaoqi Lei, Yu Wang, Gaobin Pu, Zhenhua Liu, Xue Chen, Qian Liu

**Affiliations:** College of Pharmacy, Shandong University of Traditional Chinese Medicine, Jinan, Shandong, China; LiShizhen College of Traditional Chinese Medicine, Huanggang Normal University, Huanggang, Hubei, China

**Keywords:** HD-Zip I family, *Salvia miltiorrhiza*, Abiotic stresses, Expression analysis, SmHD-Zip12, Tanshinone

## Abstract

**Background:**

The homeodomain-leucine zipper I (HD-Zip I) transcription factor is a plant-specific protein that plays an essential role in the abiotic stress response of plants. Research on the HD-Zip I family in *Salvia miltiorrhiza* is still lacking.

**Methods and Results:**

In this study, a total of 25 SmHD-Zip I proteins were identified. Their characterizations, phylogenetic relationships, conserved motifs, gene structures, and cis-elements were analyzed comprehensively using bioinformatics methods. Expression profiling revealed that *SmHD-Zip I* genes exhibited distinctive tissue-specific patterns and divergent responses to ABA, PEG, and NaCl stresses. *SmHD-Zip12* responded the most strongly to ABA, PEG, and NaCl, so it was used for transgenic experiments. The overexpression of *SmHD-Zip12* significantly increased the content of cryptotanshinone, dihydrotanshinone I, tanshinone I, and tanshinone IIA by 2.89-fold, 1.85-fold, 2.14-fold, and 8.91-fold compared to the wild type, respectively. Moreover, in the tanshinone biosynthetic pathways, the overexpression of *SmHD-Zip12* up-regulated the expression levels of *SmAACT*, *SmDXS*, *SmIDS*, *SmGGPPS*, *SmCPS1*, *SmCPS2*, *SmCYP76AH1*, *SmCYP76AH3*, and *SmCYP76AK1* compared with the wild type.

**Conclusions:**

This study provides information the possible functions of the HD-Zip I family and lays a theoretical foundation for clarifying the functional mechanism of the *SmHD-Zip12* gene in regulating the synthesis of tanshinone in *S. miltiorrhiza*.

## Introduction

Transcription factors act as key components of transcriptional regulatory mechanisms, controlling gene expression in almost all processes of plant life (Franco-Zorrilla & Solano, 2017; Romani & Moreno, 2021; Manna et al., 2021). The homeodomain-leucine zipper I (HD-Zip I) is a plant-specific transcription factor that plays an important role in a plant’s response to diversity of stresses (Henriksson et al., 2005; Li et al., 2019c). HD-Zip I contains a highly-conserved homology domain (HD) and a leucine zipper (LZ) domain (Ariel et al., 2007; Bürglin & Affolter, 2016); the former can specifically bind to DNA and the latter serves as a dimerization motif (Ruberti et al., 1991; Elhiti & Stasolla, 2009). The HD-Zip I gene family has been extensively studied in plants, such as *Arabidopsis thaliana* (Henriksson et al., 2005), *Nicotiana tabacum* (Li et al., 2019b), *Cucumis sativus* (Liu et al., 2013), *Sesamum indicum* (Mehmood et al., 2022), *Solanum lycopersicum* (Zhang et al., 2014), and *Chrysanthemum morifolium* (Song et al., 2016).

HD-Zip I proteins are involved in plant growth and development and are associated with abiotic stress responses, such as drought stress, exposure to abscisic acid (ABA), and salinity stress (Johannesson et al., 2003; Hu et al., 2012; Shen et al., 2019; Gong et al., 2019; Hussain et al., 2021; Sharif et al., 2021). For example, *ATHB7* and *ATHB12* were strongly induced by water-deficit and ABA in *A. thaliana* (Ariel et al., 2007). In *Oryza sativa*, both the expression of *OsSLI1* and *Oshox22* were induced very quickly and dramatically by ABA, PEG, and salt treatment (Zhang et al., 2012; Huang et al., 2014). Studies have also shown that the overexpression of *TaHDZipI-5* in bread wheat, encoding a stress-responsive HD-Zip I, increased the drought and frost tolerance of transgenic wheat lines (Yang et al., 2018). The overexpression of *CaHDZ12* in tobacco enhanced the plant’s tolerance to osmotic stresses and sensitivity to ABA, and knocking out *CaHDZ12* in chickpea increased the plant’s sensitivity to salt and drought stresses (Sen et al., 2017). In sunflowers, the *HD-Zip I* gene, *HaHB11*, responded to drought and salinity tolerance by closing stomata more rapidly and the plant lost less water because of this quick response (Cabello et al., 2017). The overexpression of the HD-Zip I family member, *MdHB-7*, ameliorated the restricted photosynthetic capacity and root damage caused by salt stress in apple (Zhao et al., 2021). Moreover, it has been reported that *HD-Zip I* genes mediate abiotic stress responses through ABA-mediated signal transduction pathways (Valdés et al., 2012; Zhang et al., 2012; Hussain et al., 2021). *ZmHDZ10* overexpression in *Arabidopsis* positively regulated drought and salt tolerance, likely due to the expression of stress/ABA-responsive genes, including *P5CS1*, *RD22*, *RD29B*, and *ABI1* (Zhao et al., 2014). In contrast, the overexpression of *ZmHDZ1* reduced tolerance to salt stress and increased sensitivity to exogenous ABA, suggesting that *ZmHDZ1* functions as a negative regulator in response to salt stress (Wang et al., 2017b). Although some HD-Zip I members have been functionally studied in plants, the response of the HD-Zip I gene family to abiotic stress in *S. miltiorrhiza* has not yet been studied.

*S. miltiorrhiza* is a well-known traditional Chinese medicine and is considered a model organism for medicinal plant research (Lu et al., 2020; Wang et al., 2021). Tanshinones are one of the pharmacological compounds in *S. miltiorrhiza* and have been shown to have a variety of antioxidant, antitumor, and antivirus properties (Su et al., 2015). Tanshinones are type of diterpene derived from isopentenyl diphosphate (IPP) and their isomer dimethylallyl diphosphates (DMAPP) synthesize through two different pathways in separate compartments: the mevalonate pathway (MVA pathway) in the cytosol and the methylerythritol phosphate pathway (MEP pathway) in the plastids (Jiang, Gao & Huang, 2019). An increasing number of medicines and health products use *S. miltiorrhiza* as a main ingredient, giving rise to increased market demand. However, the growth of *S. miltiorrhiza* has been seriously limited by drought, salinity, and other environmental stresses, decreasing the natural production of tanshinones to levels that cannot meet the clinical demand (Liu et al., 2011b; Wu et al., 2014; Wei et al., 2017; Jiang, Gao & Huang, 2019; Chen et al., 2021; Yu et al., 2021; Wang & Li, 2022). Therefore, research on the biosynthesis and regulation of tanshinones in *S. miltiorrhiza* is becoming increasingly relevant.

In this study, we identified 25 *SmHD-Zip I* genes. Various characteristics of these genes were analyzed, including the physicochemical parameters, phylogenetic relationships, conserved motifs, gene structure, and cis-element. Additionally, the expression patterns of *SmHD-Zip I* genes were analyzed in different tissues and ABA, PEG, and NaCl stress. Using these results, we investigated the functions of *SmHD-Zip 12* in the hairy root of *S. miltiorrhiza* through transgenic experiments. This study provides useful information on *HD-Zip I* genes to serve as basis for further research into the functions of these genes.

## Materials & Methods

### Genome-wide identification of HD-Zip I family

The amino acid sequences of HD-Zip I proteins in *A. thaliana* were used as a query probe to search the *S. miltiorrhiza* genome by Blastp. Then, the Hidden Markov Model (HMM) profile of the homeodomain (PF00046) and the leucine zipper domain (PF02183) were downloaded from the PFAM database and used for the local HMM search using HMMER3.3 (Arce et al., 2011). The HD and LZ domain of *S. miltiorrhiza* HD-Zip I was confirmed using the Conserved Domain Database (CDD; https://www.ncbi.nlm.nih.gov/cdd/?term=).

### Sequence analysis of SmHD-Zip I

The ExPASy server (Gasteiger et al., 2005) was used to predicate the physicochemical properties of SmHD-Zip I family members, including protein length, protein molecular weight (MW), theoretical isoelectric point (pI), instability index, aliphatic index, and grand average of hydropathicity (GRAVY). Then, subcellular localization was predicted according to the Plant-Ploc server (Chou & Shen, 2008).

### Phylogenetic tree, motif distribution, gene structure analysis, and promoter cis-element analysis

The amino acid sequences of HD-Zip I proteins in *A. thaliana* were downloaded from the Arabidopsis Information Resource (TAIR) database (https://www.arabidopsis.org/). The amino acid sequences of HD-Zip I proteins in *N. tabacum*, *O. sativa*, *Zea mays*, and *S. indicum* were downloaded from the Plant Transcription Factor Database (PlantTFDB; http://planttfdb.gao-lab.org/). The phylogenetic tree of HD-Zip I proteins was constructed in MEGA-X using the neighbor-joining(NJ) method with 1,000 bootstrap replications (Kumar et al., 2018). The online software Multiple EM for Motif Elicitation (MEME; https://meme-suite.org/meme/) with a total of 20 motifs was used to investigate the conserved motifs of the SmHD-Zip I (Li et al., 2019b). The gene structure of all genes was displayed using the Gene Structure Display Server (GSDS; Hu et al., 2015). About 2,000 bp of the genomic DNA sequence of *SmHD-Zip I* genes, upstream of the starting codon (ATG), were selected for the analysis of cis-elements in the promoter using the Plant Care website (Lescot, 2002).

### Plant materials and stress treatments

The *S. miltiorrhiza* cultivar ‘Huadan 2’ was used in this study. To analyze tissue-specific gene expressions, four tissues, including the root, stem, leaf, and flower, were collected from two-year-old *S. miltiorrhiza* plants at Shandong University of Traditional Chinese Medicine Medicinal Botanical Garden. The hairy roots of *S. miltiorrhiza* were used for abiotic stress tests. The hairy roots were cultured in 50 mL of liquid 6,7-V medium on an orbital shaker (Shandong, China) running at 120 rpm and then kept at 25 °C in darkness. The 18-day-old hairy roots were then treated with 100 µmol/L ABA, 20% PEG-6000, and 200 mmol/L NaCl, respectively. Samples were harvested at 0, 3, 6, and 9 h after treatments (Zhang et al., 2012; Bai et al., 2020). The samples were immediately frozen in liquid nitrogen and stored at −80 °C for RNA extraction. All treatments were replicated with at least three independent biological experiments.

### RNA extraction and expression of *SmHD-Zip I* genes

Total RNA was isolated from all samples using the FastPure Plant Total RNA Isolation Kit (Vazyme, China); 1 µg of total RNA was used for reverse-transcription into the first strand of cDNA using the PrimeScript™ RT reagent Kit with gDNA Eraser (TaKaRa, China). The TB Green Premix Ex Taq (TaKaRa, China) was used for real-time PCR according to the manufacturer’s instructions, and qRT-PCR was performed using a CFX96 Real-Time System (Bio-Rad, USA). The *β*-*actin* gene was chosen as the reference gene. Primers were designed using Primer3 and the NCBI database was used to identify their specificity. The primers used in this study are listed in Table S1. Relative expression was calculated using by the 2^−ΔΔCt^ method (Schmittgen & Livak, 2008).

### Subcellular localization of SmHD-Zip12

To determine the subcellular localization of SmHD-Zip12, the open reading frame (ORF) of SmHD-Zip12 was cloned into the pMDC202-GFP vector to form the pMDC202-SmHD-Zip12-GFP recombinant vector. The empty GFP vector and the fusion vector were transferred to *Agrobacterium tumefaciens* GV3101 and injected into tobacco leaf cells using a needleless syringe, respectively. The fluorescent signal was observed under the laser confocal microscope after two days of culture in the injected tobacco leaves under low light.

### Plant expression vector construction

To produce the overexpression constructs of *SmHD-Zip12*, the ORF sequence was amplified and cloned into the pMDC202 vector using homologous recombination. The recombinant plasmids were transformed into *Agrobacterium tumefaciens* Ar.Qual, which was then introduced into *S. miltiorrhiza* leaf explants to obtain transgenic or wild type hairy roots. Four independent lines were used in these experiments for gene expression and metabolite analyses.

### Determination of tanshinones contents

The hairy roots of the same transformation period were cultured in the 6,7-V liquid medium, taken out after 30 days of growth, and flash-frozen in liquid nitrogen at −80 °C for a high-performance liquid chromatography (HPLC) analysis. The extraction of tanshinones was conducted following the methods described by Li et al. (2019a) with modification. The hairy roots were freeze-dried for 48 h, then 0.25 g powdered hairy root was placed in a 50 mL centrifuge tube, and 25 mL methanol was added. The sample was then subjected to ultrasound for 50 min, at 100 W, 100 Hz, 50 °C and then centrifuged at 4,000 rpm for 10 min. The supernatant was removed and filtered through a 0.22 µm membrane, and the tanshinone content was measured using HPLC.

### Data statistics and analysis

The statistical analysis was performed using IBM’s SPSS Statistics 22 software and the Student’s *t*-test. All experiments were repeated in triplicate. Significant differences were considered at *p* < 0.01 and *p* < 0.05.

## Results

### Identification of SmHD-Zip I

A total of 25 HD-Zip I proteins, containing the complete HD and LZ domains, were obtained from the *S. miltiorrhiza* genome and named SmHD-Zip1 to SmHD-Zip25. All these nucleotide sequences were uploaded to NCBI with GenBank accession numbers. The amino acid sequence length of SmHD-Zip I was found to vary from 121 aa (SmHD-Zip 1) to 471 aa (SmHD-Zip 10), with an average length of 239 aa. The predicted MW of the SmHD-Zip I proteins ranged from 13.89 to 53.52 kDa. The predicted pI of the SmHD-Zip I proteins ranged from 4.48 (SmHD-Zip 8) to 9.85 (SmHD-Zip 17). An analysis of the protein instability index showed that SmHD-Zip I proteins were unstable. The aliphatic index of the protein sequences ranged from 55.24 (SmHD-Zip 6) to 79.75 (SmHD-Zip 1). The predicted GRAVY of SmHD-Zip I proteins revealed hydrophilicity. The subcellular localization prediction indicated that five SmHD-Zip I proteins were localized to the chloroplast, whereas the remaining SmHD-Zip I proteins were located in the nucleus (Table S2).

### Phylogenetic analysis of SmHD-Zip I

**Figure 1:**
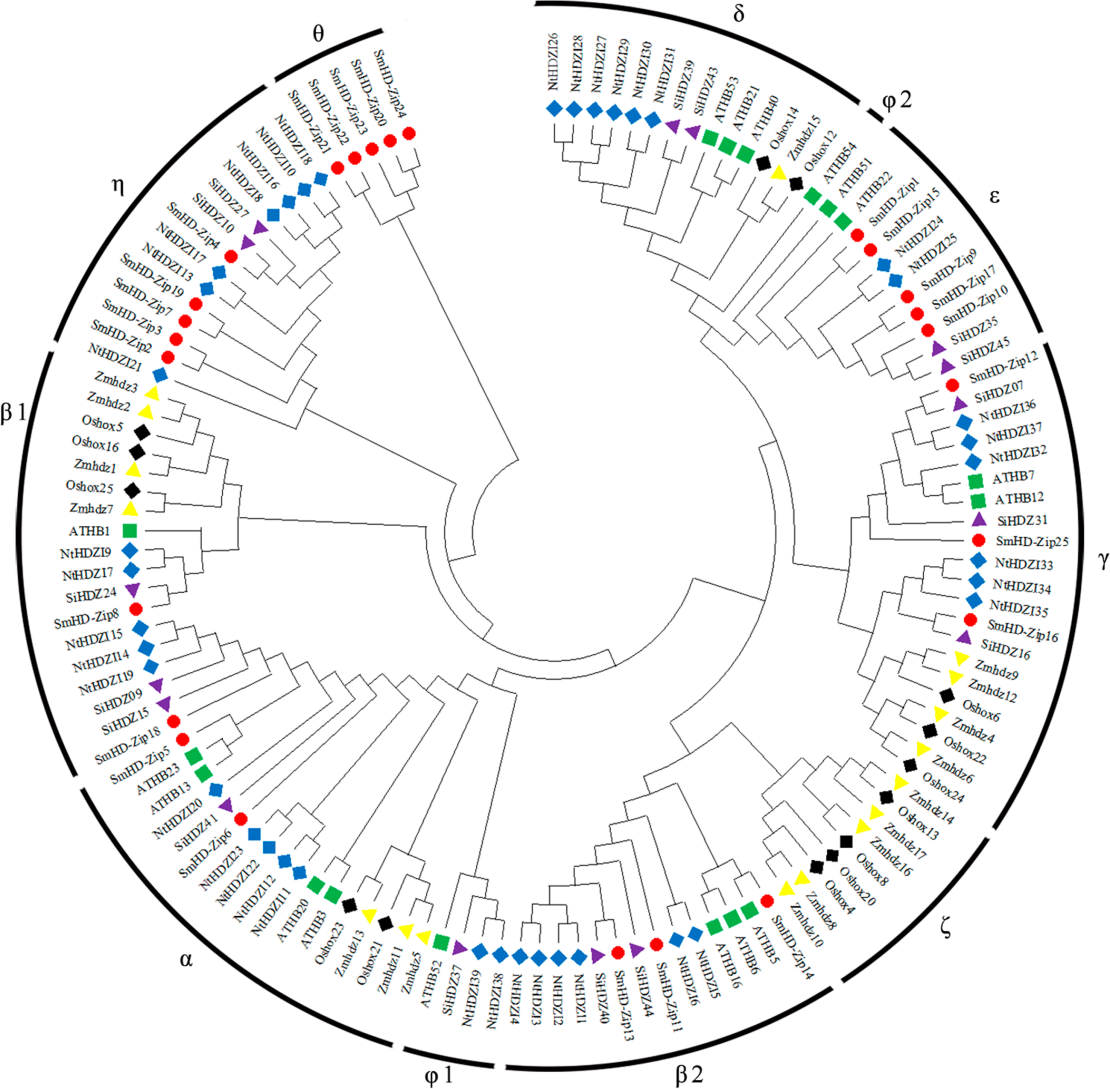
Phylogenetic tree of HD-Zip I proteins among *S. miltiorrhiza, A. thaliana, N. tabacum, O. sativa, Z. mays*, and *S. indicum*. The phylogenetic tree was generated using the neighbor-joining (NJ) method in MEGA X with 1,000 bootstrap replicates. Proteins from S. *miltiorrhiza, A. thaliana, N. tabacum, O. sativa, Z. mays*, and *S. indicum* are denoted by red circles, green squares, blue diamonds, black diamonds, yellow triangles, and purple triangles, respectively.

A phylogenetic tree was constructed of the sequence information of *S. miltiorrhiza*, *A. thaliana*, *N. tabacum*, *O. sativa*, *Z. mays*, and *S. indicum* HD-Zip I proteins (Table S3), to reveal the evolutionary relationships between the proteins. All HD-Zip I proteins were divided into 11 clades: *α*, *β*1, *β*2, *γ*, *δ*, *ɛ*, *ζ*, *η*, *θ*, *φ*1, and *φ*2 (Henriksson et al., 2005; Zhao et al., 2011; Li et al., 2019b; Fig. 1). SmHD-Zip I proteins were found in clades *α*, *β*1, *β*2, *γ*, *ɛ*, and *η*. Clades *ɛ*, *η*, and *θ* each contained five proteins. Clade *β*1 only contained the SmHD-Zip8 protein. These results provide some evidence for the functional prediction of HD-Zip I proteins in *S. miltiorrhiza*.

### Gene structure and conserved motifs analysis

**Figure 2:**
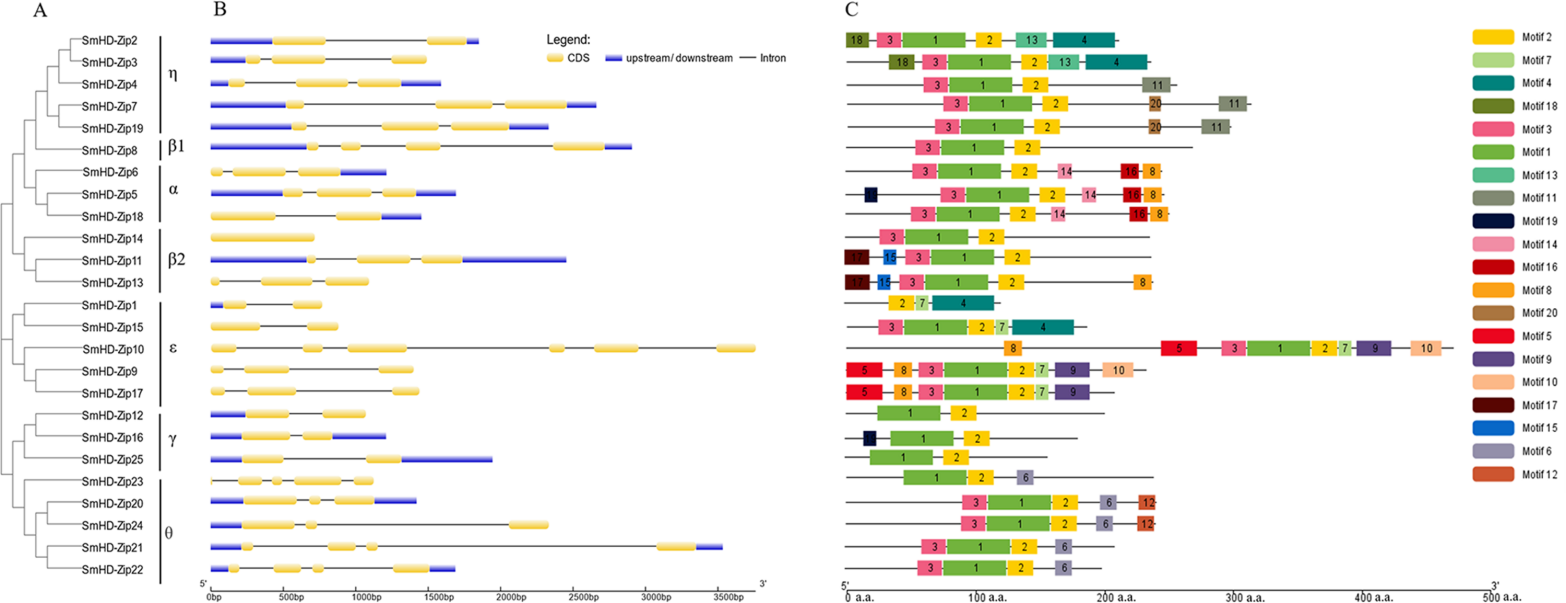
Phylogenetic relationships (A), gene structure (B), and conserved motifs (C) in SmHD-Zip I from *S. miltiorrhiza*. (A) Phylogenetic tree of SmHD-Zip I. (B) Intron and exon structures of SmHD-Zip I. Yellow boxes indicate exons, blue boxes indicate untranslated 5′-and 3′-regions, and black lines indicate introns. (C) Conserved motifs. The motifs are displayed in different colored boxes. The sequence information for each motif is provided in Table S4.

To get insight into the structural diversity of *SmHD-Zip I* genes, the exon-intron structures were analyzed using GSDS online server. The results showed that the number of introns per *SmHD-Zip I* gene varied from one to five, except for *SmHD-Zip14*, which had no intron (Fig. 2B). *SmHD-Zip10* contained the largest number of introns (5). Members of clades *α* and *η* had one to two introns, members of clade *ɛ* contained two to four introns, and member of clade *γ* contained one intron.

The conserved motifs of the SmHD-Zip I proteins were analyzed and identified using the MEME online service. The predictive analysis showed that all 25 SmHD-Zip I proteins except SmHD-Zip1, SmHD-Zip12, SmHD-Zip16, SmHD-Zip23, and SmHD-Zip25, have motifs 1, 2, and 3. The results suggested that motif 1 and motif 3 encoded the HD domain, and motif 2 encoded the LZ domain (Fig. 2C, Table S4). In general, HD-Zip I protein members of the same clade possessed similar motifs. Members of clade *α* displayed motifs 8, 14, and 16, with SmHD-Zip5 additionally containing motif 19. Members of clade *β*2, SmHD-Zip11 and SmHD-Zip13, contained motif 15 and motif 17, with SmHD-Zip13 additionally containing motif 8. Motif 6 was displayed in clade *θ* and motif 7 was displayed in clade *ɛ*.

### Cis-element analysis in the promoter region of *SmHD-Zip I* genes

To elucidate the possible regulatory mechanism of *SmHD-Zip I* genes, we used the Plant Care website to analyzed the promoter sequence of 2,000 bp of the genomic DNA sequence of *SmHD-Zip I* genes upstream of the starting codon (ATG; Table 1). We identified a total of 20 cis-elements. Among these cis-elements, six were responsive to hormones, four were responsive to stress, three were related to growth, and seven were associated with light responses. Among the hormone-related elements identified, ABRE (ABA-responsiveness) had the largest number, followed by CGTCA-motif (MeJA-responsiveness) and TGACG-motif (MeJA-responsiveness). TCA-element (SA-responsiveness), TGA-element (IAA-responsiveness), and P-box (IAA-responsiveness) were also found in the analyzed upstream region of the *SmHD-Zip I* gene. Many elements were identified that are related to stress response, including MBS, which is an MYB-binding site related to drought induction; 12 genes contained 1-2 MBS. ARE, LTR, and TC-rich repeats were also observed in the promoter region of the *SmHD-Zip I* gene. A few *SmHD-Zip I* genes contained growth-related elements, such as CAT-box (involved in meristem expression), circadian (involved in circadian rhythm control), and HD-Zip I (involved in differentiation of the palisade mesophyll cells). Light-responsive elements were also identified in the promoter region of all 25 *SmHD-Zip I* genes, except for the *SmHD-Zip17* and *SmHD-Zip19* gene, which contained the Box4 element. The distribution of these cis-elements in the promoter region of the *SmHD-Zip I* genes suggested that gene expression could be regulated at different growth stages and in response to environmental stimuli.

**Table 1:** Analysis of cis-elements in *SmHD-Zip I* genes.

**Functional** **class**	**Cis-element**	**Correlation**	**1**	**2**	**3**	**4**	**5**	**6**	**7**	**8**	**9**	**10**	**11**	**12**	**13**	**14**	**15**	**16**	**17**	**18**	**19**	**20**	**21**	**22**	**23**	**24**	**25**
Hormone	ABRE	ABA-responsiveness	–	1	3	1	3	1	1	2	0	6	3	6	0	3	2	11	0	1	3	2	0	7	4	4	3
	CGTCA-motif	MeJA-responsiveness			3	2	5	0	1	3	1	1	2	5	1	2	1	2	4	0	0	3	1	0	0	0	2	3
	TGACG-motif	MeJA-responsiveness			3	2	5	0	1	3	1	1	2	0	1	2	1	2	4	0	0	3	1	0	0	0	2	3
	TCA-element	SA-responsiveness			1	1	2	2	0	1	3	0	1	0	1	1	1	2	0	0	0	3	0	0	0	0	0	0
	TGA-element	IAA-responsiveness			0	0	2	1	0	0	0	2	0	2	0	0	0	0	1	0	0	0	0	0	0	0	1	1
	P-box	GA-responsiveness		0	0	2	1	0	0	0	0	0	0	1	0	0	1	0	0	1	0	0	1	0	0	0	0
Stress	ARE	anaerobic induction	–	3	4	1	0	3	0	3	0	1	0	1	1	1	0	2	0	1	2	2	0	0	1	1	0
	LTR	low-temperature responsiveness			2	3	0	1	3	1	0	0	0	0	1	1	4	0	1	0	0	0	0	0	0	2	0	0
	MBS	MYB binding site involved in drought-inducibility			0	0	0	3	1	1	1	0	0	1	1	2	1	2	2	0	1	0	0	0	1	0	0	0
	TC-rich repeats	defense and stress responsiveness		0	0	0	0	2	0	0	0	1	0	0	0	1	1	0	0	1	0	1	1	0	0	1	0
Development	CAT-box	meristem expression	–	1	0	0	0	0	1	1	0	0	1	0	4	1	0	1	0	0	0	0	0	0	0	0	1
	circadian	circadian control			0	0	0	0	0	0	1	0	1	0	0	1	1	1	2	0	0	0	0	0	1	0	1	0
	HD-Zip 1	differentiation of palisade mesophyll cells		0	0	1	0	0	0	0	0	1	0	0	0	2	0	0	0	0	0	1	0	0	0	0	0
Light	Box 4	light responsiveness	–	11	7	1	4	9	5	4	1	4	5	1	8	2	3	6	0	6	0	8	2	9	5	12	4
	G-Box	light responsiveness			0	2	0	0	1	0	1	0	6	2	2	0	1	0	5	0	0	1	1	0	2	2	1	1
	G-box	light responsiveness			0	0	3	2	1	1	0	0	0	1	3	0	2	1	5	0	2	1	2	0	6	2	2	4
	GT1-motif	light responsiveness			0	0	0	1	4	0	2	3	1	1	2	0	0	0	1	0	3	0	0	1	2	0	0	1
	I-box	light responsiveness		0	0	1	0	0	1	1	0	0	0	2	0	0	1	0	0	0	1	0	1	1	1	2	0
	MRE	light responsiveness		1	0	0	0	3	0	1	0	0	1	0	0	0	0	0	0	0	1	0	0	0	0	0	0
	TCT-motif	light responsiveness		0	2	2	1	1	1	0	1	1	0	0	0	0	5	2	0	1	0	1	1	1	0	3	2

### Expression profiles of *SmHD-Zip I* genes in different tissues

**Figure 3:**
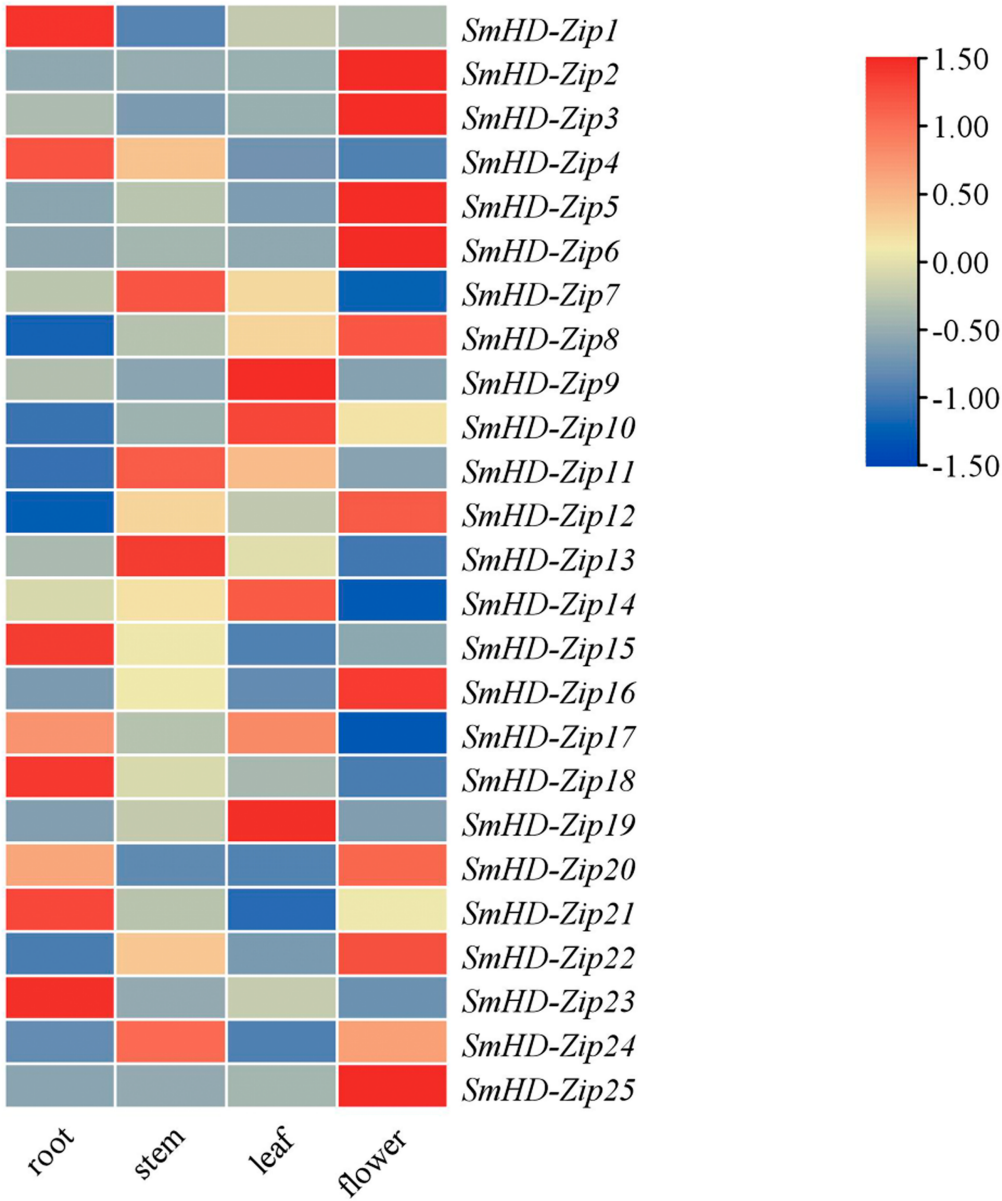
Expression analysis of the *HD-Zip I* genes in *S. miltiorrhiza*. The 2^−ΔΔCT^ method was used to calculate relative expression. Heatmap of the expression of *SmHD-Zip I* in different tissues, including the root, stem, leaf, and flower. –1.50 to 1.50 were artificially set with the color scale limits according to the normalized value. The gradient from blue to red represents low to high expression.

To study the potential biological functions of the *SmHD-Zip I* genes, we analyzed the expression profiles of *SmHD-Zip I* genes in different tissues, including the root, stem, leaf, and flower by qRT-PCR. As shown in Fig. 3, *SmHD-Zip1*, *SmHD-Zip4*, *SmHD-Zip15*, *SmHD-Zip18*, *SmHD-Zip21*, and *SmHD-Zip23* genes had the highest expression levels in the root; *SmHD-Zip7*, *SmHD-Zip11*, *SmHD-Zip13*, and *SmHD-Zip24* genes had the highest expression levels in the stem; *SmHD-Zip9*, *SmHD-Zip10*, *SmHD-Zip14*, *SmHD-Zip17*, and *SmHD-Zip19* genes had the highest expression level in leaf; and *SmHD-Zip2*, *SmHD-Zip3*, *SmHD-Zip5*, *SmHD-Zip6*, *SmHD-Zip8*, *SmHD-Zip12*, *SmHD-Zip16*, *SmHD-Zip20*, *SmHD-Zip22*, and *SmHD-Zip25* genes had the highest expression levels in the flower. The results indicated that *HD-Zip I* genes might play an important function in plant growth and development.

### Expression analysis of *SmHD-Zip I* genes in response to ABA, PEG, and NaCl

To investigate whether *SmHD-Zip I* genes respond to abiotic stress, the expression patterns of *SmHD-Zip I* genes were analyzed in the hairy root of *S. miltiorrhiza* under different stress conditions including ABA, PEG, and NaCl.

**Figure 4:**
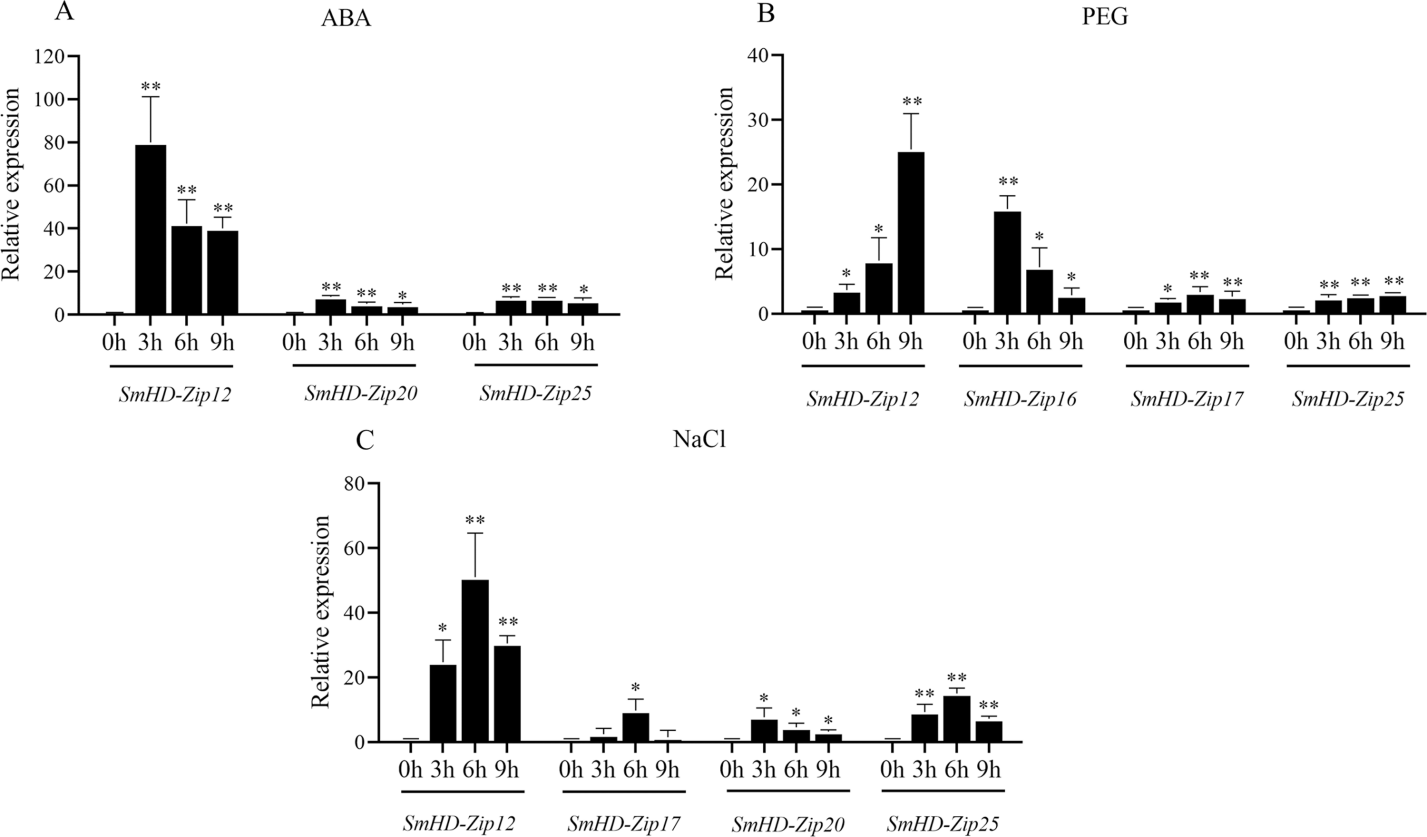
The expression patterns of *SmHD-Zip I* at 0, 3, 6, and 9 h after ABA (A), PEG (B), and NaCl (C) treatment. Hairy roots were cultured in a 6,7-V liquid medium for 18 days before being treated. The 2^−ΔΔCT^ method was used to calculate relative expression. *SmActin* was used as the internal standard. Asterisk (** and *) indicate significant differences compared to the control (0 h) at *p* < 0.01 and *p* < 0.05, respectively.

As shown in Fig. 4A, the expression levels of three *SmHD-Zip I* genes (*SmHD-Zip12, SmHD-Zip20, and SmHD-Zip25*) significantly increased, by more than 3-fold, at 3 h, 6 h, and 9 h post-ABA treatment compared to the control. *SmHD-Zip20* and *SmHD-Zip25* expression reached a maximum level at 3 h that was more than 7-fold higher than the control. The *SmHD-Zip12* gene responded to ABA the most strongly, with expression levels 80.00-fold higher than the control at 3 h post-ABA treatment. Compared with expression levels 3 h post-ABA treatment, the expression levels of *SmHD-Zip12*, *SmHD-Zip20*, and *SmHD-Zip25* declined continuously at 6 h and 9 h post-ABA treatment, but remained higher than the control. At 9 h, the expression levels of the *SmHD-Zip12*, *SmHD-Zip20*, and *SmHD-Zip25* were 40.01-fold, 4.74-fold, and 6.58-fold higher than the control, respectively. The expression levels of the genes of the other 22 *SmHD-Zip I* genes only slightly increased under ABA treatment, with expression levels less than 3-fold the control at all time points (Fig. S1A).

As shown in Fig. 4B, the expression levels of *SmHD-Zip12* and *SmHD-Zip16* significantly increased, by 3.66-fold and 16.18-fold, respectively, at 3 h post-PEG treatment compared to the control. At 6 h post-PEG treatment, *SmHD-Zip12*, *SmHD-Zip16*, and *SmHD-Zip17* expression levels increased more than 3-fold compared to the control. At 9 h post-PEG treatment, the expression of the *SmHD-Zip12* gene continued to increase to levels 25.43-fold higher than the control. The expression level of *SmHD-Zip25* increased 3.16-fold compared to the control at 9 h. The expression levels of the genes of the other 21 *SmHD-Zip I* genes only slightly increased under PEG treatment, with expression levels less than 3-fold the control at all time points (Fig. S1B).

As shown in Fig. 4C, the expression levels of three genes (*SmHD-Zip12*, *SmHD-Zip20*, and *SmHD-Zip25*) significantly increased, by more than 3-fold, at 3 h post-NaCl treatment compared to the control. The expression levels of *SmHD-Zip20* and *SmHD-Zip25* increased 7.72-fold and 9.27-fold, respectively. *SmHD-Zip20* reached its highest expression level at 3 h. At 3 h post-NaCl treatment, the expression level of *SmHD-Zip 12* was 24.72-fold higher than the control. At 6 h post-NaCl treatment, the expression level of the *SmHD-Zip12* gene reached a level 51.00-fold higher than the control. *SmHD-Zip17* and *SmHD-Zip25* expression peaked at 6 h post-NaCl treatment with expression levels more than 7-fold and 15-fold higher than the control, respectively. At 9 h post-NaCl treatment, the expression levels of *SmHD-Zip12*, *SmHD-Zip20*, and *SmHD-Zip25* were lower compared to 6 h, but remained higher than the control. The expression level of the genes of the other 21 *SmHD-Zip I* genes only slightly increased under NaCl treatment, with expression levels less than 3-fold the control at all time points (Fig. S1C).

These results indicate that the *SmHD-Zip 12* gene quickly and strongly responds to ABA, PEG, and NaCl.

### Subcellular localization of SmHD-Zip12

**Figure 5:**
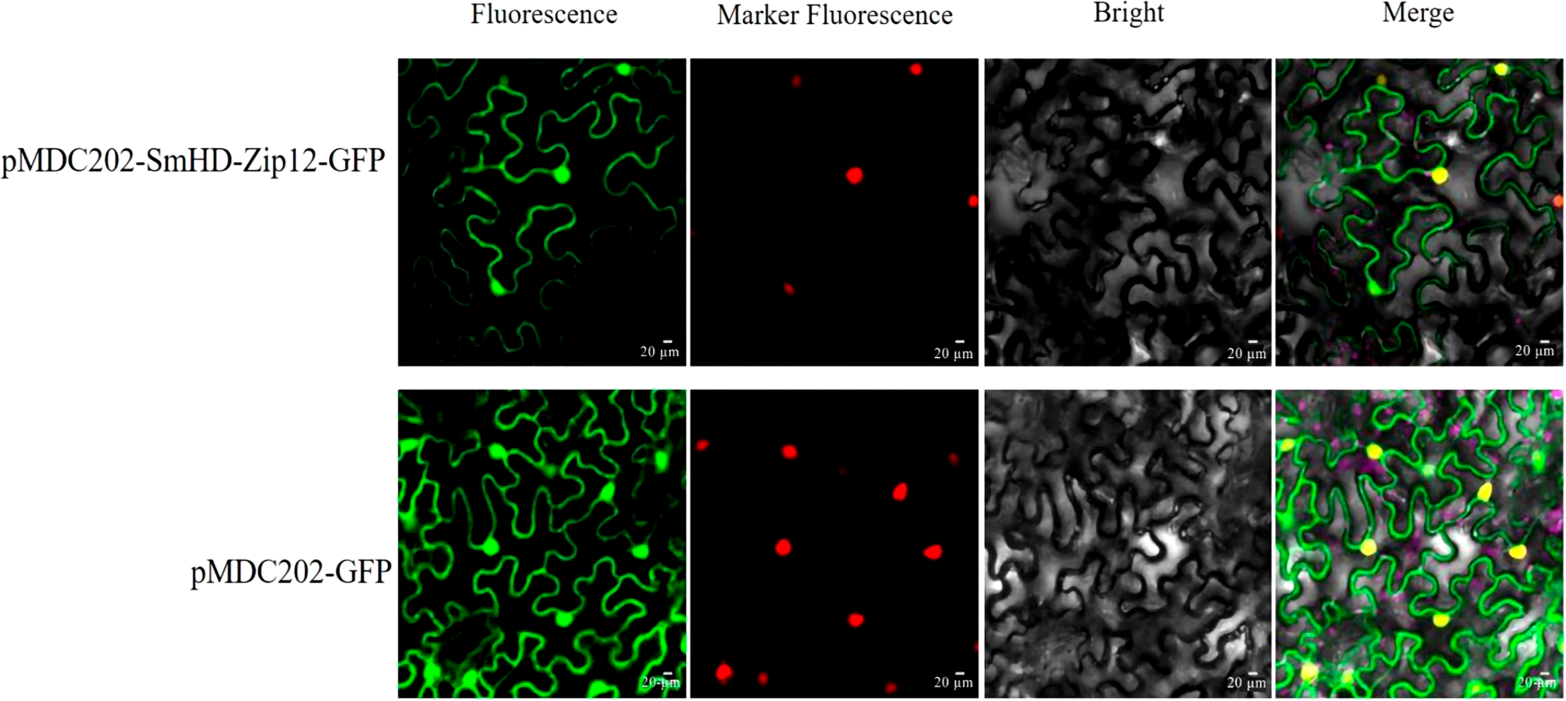
Subcellular localization analysis of SmHD-Zip12 in tobacco leaf cells. The pMDC202-SmHD-Zip12-GFP (upper lane) and pMDC202-GFP (bottom lane) plasmids were transiently expressed in tobacco leaf cells. Fluorescence was observed using a confocal laser scanning microscope. The pictures show fluorescence, nucleus fluorescence, bright, and merge of four fields from left to right.

Generally, the physiological function of a protein is linked to its intracellular location. The protein subcellular localization prediction result showed that the SmHD-Zip12 protein was localized in the nucleus (Table S2). To verify this prediction for the SmHD-Zip12 protein, the fusion protein vector pMDC202-SmHD-Zip12-GFP was constructed. As shown in Fig. 5, tobacco leaf epidermal cells with pMDC202-SmHD-Zip12-GFP fusion protein showed fluorescence signals only in the nucleus under microscope, whereas the control GFP protein was diffused throughout the cell, suggesting that SmHD-Zip12 is a nuclear-localized protein.

### *SmHD-Zip12* was involved in the tanshinone biosynthesis

To further study the functions of *SmHD-Zip12*, the *SmHD-Zip12* over-expressed (OE) vector was constructed and transgenic hairy root lines were obtained. The desired transgenic lines were identified by PCR using *rolB* and *SmHD-Zip12*-specific primers, with a positive rate of 70.59% (Figs. S2A and S2B). Four *SmHD-Zip12* over-expression lines(OE1, OE2, OE3, and OE4) were selected for further experiments based on *SmHD-Zip12* expression level using qRT-PCR (Fig. S2C).

**Figure 6:**
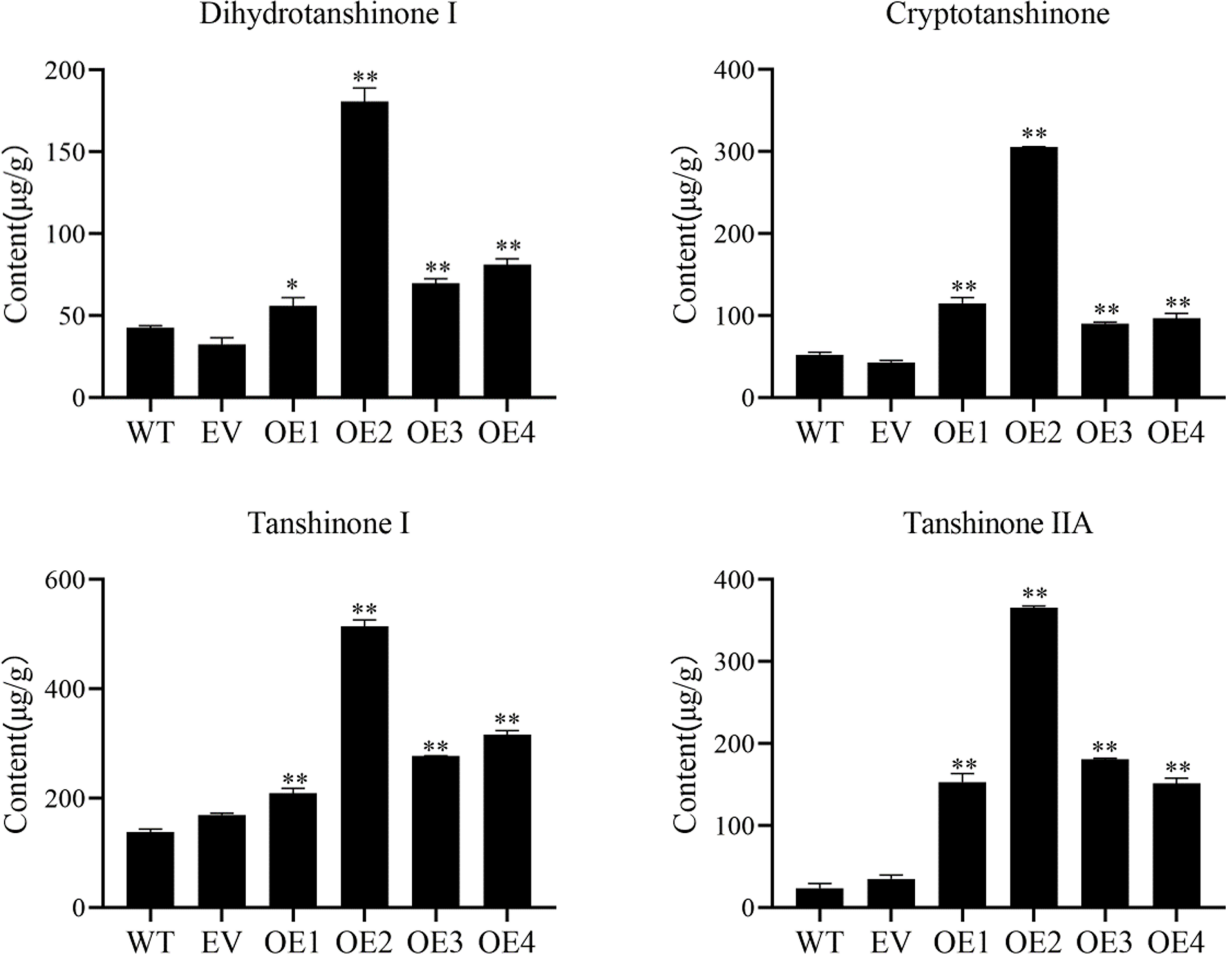
Cryptotanshinone, dihydrotanshinone I, tanshinone I, and tanshinone IIA content in hairy roots of *S. miltiorrhiza*, measured by HPLC. Tanshinone concentration in the wild type (WT), empty vector (EV), and the four independent *SmHD-Zip12*-overexpressed (OE) transgenic hairy root lines. Asterisks (** and *) indicate significant differences compared to the control (WT) at *p* < 0.01 and *p* < 0.05, respectively.

Cryptotanshinone (CT), dihydrotanshinone I (DT-I), tanshinone I (T-I), and tanshinone IIA (T-IIA) content in *SmHD-Zip12* overexpression hairy roots were measured by HPLC (Fig. 6). The results showed that the overexpression of *SmHD-Zip12* significantly increased CT, DT-I, T-I, and T-IIA contents, by 2.89-fold, 1.85-fold, 2.14-fold, and 8.91-fold, respectively compared with the wild type. In particular, the content of T-IIA in overexpression line 2 was 15.31-fold (365.48 µg/g DW) higher than the wild type (23.86 µg/g DW).

**Figure 7:**
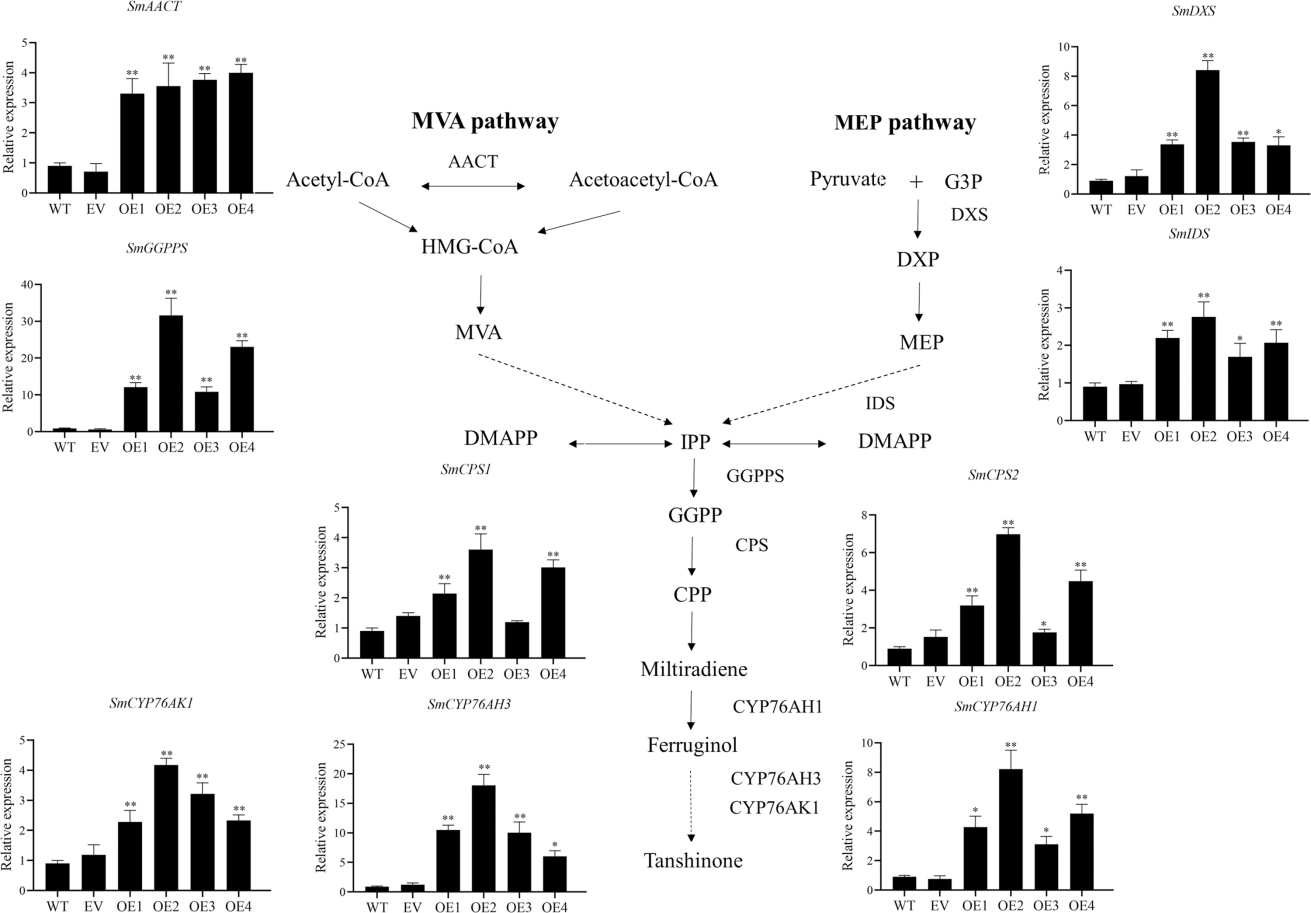
Relative expression analysis of nine tanshinone biosynthesis genes (*SmAACT, SmDXS, SmIDS, SmGGPPS, SmCPS1, SmCPS2, SmCYP76AH1, SmCYP76AH3*, and *SmCYP76AK1*) by qRT-PCR. Asterisks (** and *) indicate significant differences compared with the wild type (WT) at *p* < 0.01 and *p* < 0.05, respectively. The solid and dashed lines represent one-step and multi-step reactions, respectively.

The key enzyme genes in the biosynthesis pathway of tanshinones in overexpression *SmHD-Zip12* hairy roots were analyzed by qRT-PCR (Fig. 7). The results showed that the relative expression levels of *SmAACT*, *SmDXS*, *SmIDS*, *SmGGPPS*, *SmCPS1*, *SmCPS2*, *SmCYP76AH1*, *SmCYP76AH3*, and *SmCYP76AK1* in the overexpression lines were significantly up-regulated compared with the wild type. In particular, the expression levels of the *SmGGPPS* gene and *SmCYP76AH3* gene in overexpression line 2 were 31.63-fold and 13.16-fold higher, respectively, compared with the wild type.

## Discussion

HD-Zip I transcription factors are plant-specific proteins that play important roles in the growth of plants. There are 17 *HD-Zip I* genes in *A. thaliana* (Henriksson et al., 2005), 15 *HD-Zip I* genes in *Eucalyptus grandis* (Zhang et al., 2020), 20 *HD-Zip I* genes in *Triticum aestivum* (Yue et al., 2018), 16 *HD-Zip I* genes in *Hordeum vulgare* (Li et al., 2019d), and 30 *HD-Zip I* genes in *Glycine max* (Chen et al., 2014) , but little is known about *HD-Zip I* genes in *S. miltiorrhiza*. In this study, we identified 25 *HD-Zip I* genes in *S. miltiorrhiza* and then systematically analyzed their characteristics. Gene duplication and gene loss have resulted in different numbers of *HD-Zip I* genes during evolution (Que et al., 2018). The results of this study provide useful information for the functional characterization of SmHD-Zip I gene family.

Our conserved domain analysis showed that SmHD-Zip I proteins contained the conserved HD domain and the LZ domain, which is characteristic of HD-Zip I transcription factors (Liu et al., 2013; Zhang et al., 2014; Mehmood et al., 2022). The conserved motifs can reflect their protein-specific functions (Li et al., 2014; Liu et al., 2022). A motifs analysis showed that motifs 1 and 3 encoded the HD domain, which was involved in DNA binding (Zhang et al., 2014). Motif 2 encoded the LZ domain, which was involved in protein-protein interactions (Ruberti et al., 1991; Ariel et al., 2007). Similar results were found in *Capsicum annuum* (Zhang et al., 2021) and *Medicago truncatula* (Li et al., 2022). The evolutionary characteristics of the exon-intron structure have a role in the evolution of functions in paralogs (Boudet et al., 2001). The CDS was discontinuous due to the presence of introns. Furthermore, the loss or increase of introns may lead to changes in the length of gene introns (Zhang et al., 2020).

Cis-acting elements located upstream of transcription initiation sites control the regulation of gene expression (Hernandez-Garcia & Finer, 2014). ABRE (ABA-responsive element), as a decisive cis-element in most gene promoters, is involved in ABA regulation. ABRE belongs to the G-box family and contains an ACGT core, a sequence that is recognized by plant bZIP proteins (Gómez-Porras et al., 2007; Fujita et al., 2011). There are four ABREs in *HA-HB4*, one of which is responsible for the regulation of ABA, NaCl, and drought-related stress (Manavella et al., 2008). ABRE and MBS cis-acting elements that respond to hormones and environmental stresses were identified in the *MdHB-1* gene promoter region (Wang et al., 2017a). In this study, ABREs were found in 20 *SmHD-Zip I* genes, which were also identified in *M. truncatula* (Li et al., 2022). *TaHDZipI-3*, *TaHDZipI-4*, and *TaHDZipI-5* genes were characterized to bind to stress-related cis-elements and were differentially expressed under ABA treatment, cold treatment and water deficit (Harris et al., 2016; Mehmood et al., 2022). MBS cis-element was found in half of the *SmHD-Zip I* genes, and one to four LTR cis-elements were found in ten of the *SmHD-Zip I* genes, suggesting that *SmHD-Zip I* genes may be involved in the plant’s response to drought stress and low-temperature stress. In addition, growth-related elements and light-responsive elements were also found in *SmHD-Zip I* gene promoters. These results contribute to the understanding of the diverse transcriptional regulatory mechanisms of *SmHD-Zip I* genes in *S. miltiorrhiza*.

The HD-Zip I proteins play a role in the plant’s response to ABA and abiotic stresses (Gong et al., 2019). In *M. truncatula*, *MtHB12* was highly inducible by salt, osmotic, and ABA stresses (Li et al., 2022). Overexpression of *CaHDZ12* tobacco lines improved the plant’s tolerance to water deficit and salt stress and increased its sensitivity to ABA (Sen et al., 2017). In *A. thaliana*, *ATHB12* was induced by ABA, water deficit, and NaCl (Shin et al., 2004; Valdés et al., 2012). A T-DNA insertion mutant of *ATHB12* reduced sensitivity to ABA during plant germination (Son et al., 2010). *OsHOX24* and *OsHOX22* in rice, which are homologous genes of *ATHB12*, were highly expressed when subjected to stresses such as salinity and osmotic stress (Bhattacharjee, Khurana & Jain, 2016). *OsHOX24* participates in the regulation of abiotic stress responses by regulating the expression of stress-responsive genes in rice (Bhattacharjee, Sharma & Jain, 2017). In this study, expression levels of *HD-Zip I* genes in *S. miltiorrhiza* were also induced under different stresses. Notably, the expression of *SmHD-Zip12* increased under ABA, PEG, and NaCl stresses significantly more than the other genes. The expression level of *SmHD-Zip12* was 80.00-fold higher at 3 h post-ABA exposure, 25.43-fold higher at 9 h post-PEG exposure, and 51.00-fold higher at 6 h post-NaCl exposure than under control conditions, respectively. Based on these results, we speculated that the *SmHD-Zip12* gene participates in regulating abiotic stress.

Most transcription factors play the role of regulators *in vivo* and are often located in the nucleus (Wang et al., 2017b). GFP fusion protein in the leaves of tobacco showed that SmHD-Zip12 is localized exclusively in the nucleus, indicating that SmHD-Zip12 functions as a transcription factor. The results helped to further understand the mechanism of action of SmHD-Zip12. Studies have revealed the role of *HD-Zip I* genes in responses to abiotic stress (Manavella et al., 2006; Bhattacharjee, Sharma & Jain, 2017; Sen et al., 2017). In addition, abiotic stress could promote the accumulation of tanshinones (Zhao, Zhou & Wu, 2010; Liu et al., 2011a; Yang et al., 2012; Wei et al., 2016). Therefore, we speculated that HD-Zip I is closely related to tanshinone biosynthesis. In this study, the overexpression of *SmHD-Zip12* significantly increased the contents of CT, DT-I, T-I, and T-IIA. Most of the key enzyme genes in the tanshinone synthesis pathway have now been cloned and identified. *GGPPS* is considered an important regulatory target in the tanshinone biosynthetic pathway, and overexpression of *SmGGPPS* could increase the content of tanshinones (Kai et al., 2011; Shi et al., 2016). *CYP76AH3* plays a key role in the production of intermediates, and silencing *SmCYP76AH3* reduced the content of tanshinones (Guo et al., 2016). In this study, most of the genes, such as *SmAACT*, *SmDXS*, *SmIDS*, *SmGGPPS*, *SmCPS1*, *SmCPS2*, *SmCYP76AH1*, *SmCYP76AH3*, and *SmCYP76AK1*, were up-regulated in *SmHD-Zip12* overexpression lines, specifically the *SmGGPPS* and *SmCYP76AH3* gene. Our results demonstrated that *SmHD-Zip12* could regulate tanshinone biosynthesis through the activation of the key enzyme genes of the tanshinone synthesis pathway.

## Conclusions

In the present study, a total of 25 HD-Zip I transcription factors were identified in *S. miltiorrhiza*. A phylogenetic analysis was performed and the gene structures, motifs, cis-elements, tissue-specific expression levels, and stress-induced expression levels were determined. Notably, *SmHD-Zip12* was highly up-regulated after ABA, PEG, and NaCl stress. The over-expression of *SmHD-Zip12* promoted tanshinone accumulation and the expression of the key enzyme genes in the tanshinone synthetic pathway. These results indicate that *SmHD-Zip12* is a positive regulator of tanshinone biosynthesis in *S. miltiorrhiza*. This study provides abundant information for the future investigation of more potential *HD-Zip I* genes in *S. miltiorrhiza*.

##  Supplemental Information

10.7717/peerj.15510/supp-1 Data S1 Raw data

10.7717/peerj.15510/supp-2 Table S1 The expression patterns of the *SmHD-Zip I* at 0, 3, 6, and 9 h after treated with ABA (A), PEG (B), and NaCl (C). Hairy roots were cultured in a 6,7-V liquid medium for 18 days before being treated. The 2^−ΔΔCT^ method was used to be an evaluation of the relative expression. *SmActin* was used as the internal standard. ** and * indicate significant differences compared to the control (0 h) at *p* < 0.01 and *p* < 0.05, respectively.

10.7717/peerj.15510/supp-3 Table S2 Characteristics of SmHD-Zip I

10.7717/peerj.15510/supp-4 Table S3 Protein sequences of HD-Zip I in *Salvia miltiorrhiza*, *Arabidopsis thaliana*, *Nicotiana tabacum*, *Oryza sativus*, *Zea mays*, and *Sesamum indicum*

10.7717/peerj.15510/supp-5 Table S4 The motif sequence information of SmHD-Zip I

10.7717/peerj.15510/supp-6 Figure S1 The expression patterns of the *SmHD-Zip I* at 0, 3, 6, and 9 h after treated with ABA (A), PEG (B), and NaCl (C) Hairy roots were cultured in a 6,7-V liquid medium for 18 days before being treated. The 2^−ΔΔCT^ method was used to be an evaluation of the relative expression.* SmActin* was used as the internal standard. ** and * indicate significant differences compared to the control (0 h) at *p* < 0.01 and *textitp* < 0.05, respectively.

10.7717/peerj.15510/supp-7 Figure S2 Identification of SmHD-Zip12 overexpression hairy roots. (A)PCR identification of hairy roots by rolB primers M, DL-2000; 1, WT; 2, EV; 3-10, Overexpression of SmHD-Zip 12. (B)PCR analyses of transgenic hairy root lines by SmHD-Zip12-specific primers. M, DL-2000; N1: H2O; N2: WT-PC: Recombinant plasmid; 1–17: Overexpression of SmHD-Zip 12. (C)Relative expression analysis of SmHD-Zip 12 genes by qRT-PCR in in transgenic lines and WT. ** and * indicate significant differences compared with the wild type (WT) at *p* < 0.01 and *p* < 0.05, respectively.

## Additional Information and Declarations

### DNA Deposition

The following information was supplied regarding the deposition of DNA sequences: Sequence data is available at NCBI accession numbers: OP607147-OP607171

### Data Availability

The following information was supplied regarding data availability: The raw data are available in the Supplemental File.
