# Supplementary material for: Analysis of the HD-Zip I transcription factor family in Salvia miltiorrhiza and functional research of SmHD-Zip12 in tanshinone synthesis
Source: PeerJ. 2023 Jun 27;11:e15510. doi: 10.7717/peerj.15510 (PMC10312201; doi:10.7717/peerj.15510)
Supplement: Table S1 — Hairy roots were cultured in a 6,7-V liquid medium for 18 days before being treated. The 2−ΔΔCT method was used to be an evaluation of the relative expression. SmActin was used as the internal standard. ** and * indicate significant differences compared to the control (0 h) at p < 0.01 and p < 0.05, respectively. [file peerj-11-15510-s002.docx]

**Table S1**. Primers used to determine the expression levels of *SmHD-Zip I* genes by qRT-PCR

| **Gene name** | **Forward primer（5^，^-3^，^）** | **Reverse primer（5^，^-3^，^）** | **Objective** |
| --- | --- | --- | --- |
| *SmActin* | GGTGCCCTGAGGTCCTGTT | AGGGAACCACCGATCCAGACA | internal standard |
| *SmHD-Zip1* | CTCAAGCAGGAGTTCGACG | GCTCGGAATGGATGTGCTTT | gene expression |
| *SmHD-Zip2* | GAAATTAGCCCTCTCGACGC | TGCCAGAACCATGTTCCTTG | gene expression |
| *SmHD-Zip3* | GAGGAGAGAGAAGCAACCGA | TCCGGTTCGAGCTTGTTCTC | gene expression |
| *SmHD-Zip4* | ACGACGAATTTGACCCTTGC | AGCACCTTGGTTTTGTAGCG | gene expression |
| *SmHD-Zip5* | ACCACAAACTTCACGCTGAG | CCAAAACCCTGGCTGATCAT | gene expression |
| *SmHD-Zip6* | TGAACAAGGAAAACGAGGGC | AATTCTGCTGTTCCGACCAG | gene expression |
| *SmHD-Zip7* | ACTTCAACTGGCAAACGAGC | ATCTTGAGCAGTCCCATCGT | gene expression |
| *SmHD-Zip8* | GTCGGAGGAAGAACGGTGAT | CTTGTTCTCCGCCTCAAAGC | gene expression |
| *SmHD-Zip9* | CAGACAATGCTGCCGATGAT | CCAGCTTCATTTTCCGGTCC | gene expression |
| *SmHD-Zip10* | GCCGAGAAACCATCAACGTT | GATAGAGCCGTTCGTCCAGA | gene expression |
| *SmHD-Zip11* | GATTACGGCCTCCTCAAAGC | CGCCCATCATCTCGTTTTCC | gene expression |
| *SmHD-Zip12* | AGCGAACTGAACGGAAGAGA | TAGAAGTCCCACCACTGCG | gene expression |
| *SmHD-Zip13* | CCTTGGGTGGTTTGATGTCC | TGACCTGATCCACGCTCAAT | gene expression |
| *SmHD-Zip14* | AGATTGAGCGTGGATCAGGT | TTGGAGCCTCTCGAAGTTGA | gene expression |
| *SmHD-Zip15* | GGAGATGAAGCAGGCAATGG | GGCCAGCTTCATTTTCCGAT | gene expression |
| *SmHD-Zip16* | CTCAAATCCACCTACGACGC | TCATCACTTCCTCTCCACGT | gene expression |
| *SmHD-Zip17* | CAGACAATGCTGCCGATGAT | CCAGCTTCATTTTCCGGTCC | gene expression |
| *SmHD-Zip18* | CGGTGGAAGACAAAGCAGTT | TCCAGCTTGACATTCTCCGA | gene expression |
| *SmHD-Zip19* | CAGATTGCAGTGTGGTTCCA | TGTATCTCCACCAACGTCGT | gene expression |
| *SmHD-Zip20* | CCTTGAGCCCTCATTGACTC | TCGTCTTCGTCACTGACTCG | gene expression |
| *SmHD-Zip21* | AGACGAAGACTGCCCATACG | TCTGAAACCACACCTCCACT | gene expression |
| *SmHD-Zip22* | AGGCAAGTTGAGGTCTGGTT | GTGGACGTTAATTGCCCTGA | gene expression |
| *SmHD-Zip23* | TGCGAGAATTTGACGGAGGA | CAGAGCAGCGTGAAATCTCC | gene expression |
| *SmHD-Zip24* | AGGAAGCTACACAAGGAGGT | GGTGCCGGAGATGAGACA | gene expression |
| *SmHD-Zip25* | TGGAGAAGCTGAAGGGAAAG | GACGTCGTTTCCACCATTCT | gene expression |
| *SmHD-Zip12* | TTTGGAGAGGACCTCGACTCTAGAATGTCTACCAGTATTAAGAAGGGCACCA | TACTCATTTTTTCTACCGGTACCCCTCCACCACCGAAGTCCCACCACTGCGCA | subcellular localization |
| *SmHD-Zip12* | GAGGACCTCGACTCTAGAATGTCTACCAGTATTAAGAAGGGCACCA | CATTTTTTCTACCGGTACCCTAGAAGTCCCACCACTGCGCA | gene expression |
| *rolB* | GCTCTTGCAGTGCTAGATTT | GAAGGTGCAAGCTACCTCTC | positive identification |
| *SmHD-Zip12*-specific | GAGCACGACACACTTGTCTACT | CTGCGCAGTACCACCAGTATT | positive identification |
| *SmAACT* | TGTTCGGATGGACAAGCAGA | ACCATCACGATCCCATCTCC | gene expression |
| *SmDXS* | GACTTTCGGACTAGCAGGGT | AATCCTGCATTGTTGAGGGC | gene expression |
| *SmCPS1* | TAGCGACCCTCACTCAGTTC | GCGCCAGTATTTCTTCCCTG | gene expression |
| *SmCPS2* | GACACTTTCAACGGAGGAGC | ACTCCGATTCTCTCCCACTG | gene expression |
| *SmIDS* | ACAAGGGTGATGTGGTGGTA | GCCACAGTCTCTTCATGGGA | gene expression |
| *SmGGPPS* | TTTGTTCACGACGACTTGCC | CTGCGAGACGAGTTGAGTTG | gene expression |
| *SmCYP76AH1* | TCGTGGATGAGTCGGCAAT | TGAGTATCTGAGTTCCCT | gene expression |
| *SmCYP76AH3* | CCCCAACTTCGCCGACTACTTC | CAGCCTGGGCATGAGCGACT | gene expression |
| *SmCYP76AK1* | CCACCAACATGGGCGTTCC | TGTCGGATTCCTCCACGATGCT | gene expression |
