# Supplementary material for: Analysis of the HD-Zip I transcription factor family in Salvia miltiorrhiza and functional research of SmHD-Zip12 in tanshinone synthesis
Source: PeerJ. 2023 Jun 27;11:e15510. doi: 10.7717/peerj.15510 (PMC10312201; doi:10.7717/peerj.15510)
Supplement: Table S2 [file peerj-11-15510-s003.docx]

**Table S2.** Characteristics of SmHD-Zip I

| **Gene name** | **Amino Acid(aa)** | **MW (kDa)** | **pI** | **Instability Index** | **Aliphatic Index** | **GRAVY** | **Subcellular localization** |
| --- | --- | --- | --- | --- | --- | --- | --- |
| SmHD-Zip1 | 121 | 13.89 | 8.69 | 50.79(unstable) | 79.75 | -0.769 | Chloroplast |
| SmHD-Zip2 | 214 | 24.81 | 5.29 | 60.04(unstable) | 62.9 | -1.04 | Nucleus |
| SmHD-Zip3 | 239 | 27.42 | 4.96 | 61.04(unstable) | 62.47 | -0.946 | Nucleus |
| SmHD-Zip4 | 259 | 29.59 | 4.74 | 66.03(unstable) | 63.63 | -0.811 | Nucleus |
| SmHD-Zip5 | 250 | 28.83 | 5.82 | 54.23(unstable) | 66.36 | -0.868 | Nucleus |
| SmHD-Zip6 | 248 | 28.11 | 6.67 | 61.31(unstable) | 55.24 | -0.896 | Nucleus |
| SmHD-Zip7 | 316 | 35.64 | 5.05 | 47.77(unstable) | 59.24 | -0.816 | Nucleus |
| SmHD-Zip8 | 271 | 30.96 | 4.48 | 59.35(unstable) | 66.57 | -0.828 | Nucleus |
| SmHD-Zip9 | 233 | 26.69 | 7.01 | 54.87(unstable) | 72.49 | -0.647 | Nucleus |
| SmHD-Zip10 | 471 | 53.52 | 7.59 | 43.80(unstable) | 71.7 | -0.57 | Nucleus |
| SmHD-Zip11 | 239 | 26.87 | 5.24 | 57.62(unstable) | 70.17 | -0.902 | Nucleus |
| SmHD-Zip12 | 200 | 22.95 | 5.42 | 57.37(unstable) | 79.1 | -0.745 | Nucleus |
| SmHD-Zip13 | 240 | 27.39 | 4.64 | 63.29(unstable) | 63.42 | -1.012 | Nucleus |
| SmHD-Zip14 | 239 | 26.5 | 9 | 40.19(unstable) | 72.38 | -0.964 | Chloroplast |
| SmHD-Zip15 | 186 | 21.19 | 7.76 | 50.25(unstable) | 71.83 | -0.821 | Chloroplast |
| SmHD-Zip16 | 180 | 21.54 | 7.93 | 59.81(unstable) | 62.33 | -1.2 | Nucleus |
| SmHD-Zip17 | 208 | 24.64 | 9.85 | 55.96(unstable) | 75.53 | -0.831 | Chloroplast |
| SmHD-Zip18 | 254 | 28.99 | 7.07 | 63.24(unstable) | 70.71 | -0.836 | Nucleus |
| SmHD-Zip19 | 300 | 34.05 | 4.91 | 52.18(unstable) | 65.07 | -0.857 | Nucleus |
| SmHD-Zip20 | 241 | 27.09 | 8.6 | 50.11(unstable) | 69.67 | -0.763 | Nucleus |
| SmHD-Zip21 | 214 | 24.37 | 8.22 | 77.05(unstable) | 72.99 | -0.711 | Nucleus |
| SmHD-Zip22 | 199 | 22.75 | 7.08 | 83.5(unstable) | 63.77 | -0.83 | Nucleus |
| SmHD-Zip23 | 238 | 27.31 | 9.7 | 67.77(unstable) | 72.94 | -0.655 | Chloroplast |
| SmHD-Zip24 | 240 | 26.62 | 8.38 | 53.04(unstable) | 69.12 | -0.733 | Nucleus |
| SmHD-Zip25 | 177 | 20.43 | 5.31 | 55.83(unstable) | 66.72 | -0.89 | Nucleus |
