# Supplementary material for: Analysis of the HD-Zip I transcription factor family in Salvia miltiorrhiza and functional research of SmHD-Zip12 in tanshinone synthesis
Source: PeerJ. 2023 Jun 27;11:e15510. doi: 10.7717/peerj.15510 (PMC10312201; doi:10.7717/peerj.15510)
Supplement: Table S3 [file peerj-11-15510-s004.docx]

**Table S3.** Protein sequences of HD-Zip I in *Salvia miltiorrhiza*, *Arabidopsis thaliana*, *Nicotiana tabacum*, *Oryza sativus*, *Zea mays*, and *Sesamum indicum*

***Salvia miltiorrhiza***

>SmHD-Zip1

MKLAKELGLQPRQIAVWFQNRRARWKGKQLERLYDALKQEFDAVSREKHKLHQEVLALRAILKDQVAKKQVSTDVSGEETVESTSIPSSEKGRGEGNNQMGECNYVLNVEDYSPLIMTPYN

>SmHD-Zip2

MVSFEQSRGERSNRSRQSDCDDEDSDSCFHRPPEKKRRLTAEQVQFLEKSFAVENKLEPDRKNELAKKLGLQPRQVAIWFQNRRARSKTKVLEREYGSLKSCYDKLKADYDTLFSDNEKLKNEVRLLSGKLLQKGNDEISPLDAAPCKQEDASSHSSHVLEASDFEQGDEDDECLVQRLCLPKLEAEYYDDHLQPNSCNLGFPVQNQGTWFWQC

>SmHD-Zip3

MKSSPVFDVADALLNDERIPSDALWIPNSSPSLHAMVSFEQSRGERSNRSRQSDCDDGDSDSCFHHPPEKKRRLTAEQVQFLEKSFAVENKLEPDRKNELAKKLGLQPRQVAIWFQNRRARSKTKVLEREYGSLKSCYDKLKADYDTLFSDNEKLKNEKGNDEISPLDAAPCKQEDASSYSSHVLEASDFEQGDEDDECLVQRLCLPKLEAEYYDDHLQPNSCNLGFPVQNQGTWFWQC

>SmHD-Zip4

MEGGGVYDDSRMPNHLLKSESSEVLDSLWVPNSSPSFQGSASMVSFEGENINVKAENCNDEFDPCFQQPEKKRRLSHDQVQFLEKSFEVENKLEPERKIQLAKEVGLQPRQVAIWFQNRRARYKTKVLEKEYAALKASYDKLTSDYDALFKENDNMRNQVNLLTEKLLRKSPSAKKEEAASSAVFDSESQSATLEPADSSHVEASDFSQDEDDTLSRSLMPPSCFPKLEIECYDDLQPNSCNLGFPVQDQVQGTWFWQY

>SmHD-Zip5

MTCSTEMAFFHPNFMLQTPHQDHDHTHPSTSLPPLIPSSTPQDQYHGVGSLIGKRSNVCLEEMMKNENENVQDELSDDGSQIGEKKRRLNLEQVKTLEKNFEVGNKLEPERKMQLARALGLQPRQIAIWFQNRRARWKTKQLEKDYDLLKRQFDAVKAENDALQAHNHKLHAEIMALKSREATDLCINLNKETEGSCSENSSEIKLHISTAPPDPKLDQTVKEESLCNMFCGMDDQPGFWPWLDQQNFKF

>SmHD-Zip6

MAYVPAESMFRNHDVDAHFPSSSPQDFHGVPEFLMRRSMSFSGMDRAEDDEGSDDGSQMLGEKKRRLNLEQVKALEKSFELGNKLEPERKMQLARALGLQPRQIAIWFQNRRARWKTKQLEKDYDILKRQFEALKADNDSLKSQNKKLQNQLSGMKRRGEAGAGAGAINLNKENEGSWSNGSDTSCEVNLKASTEYNQRSKHAAAAMFPAAATLAHHSLPAECFGNMFGGIEETPQEFWPWSEQQNFH

>SmHD-Zip7

MAGREFANSGGGALDLSKKMKTCGHDRPLDSVFASAPSNSFLGPGAMVSFGAVGRDNSSGNSFYQSFEMQENGEEYLDDYFSQPEKKRRLSVDQVQFLERSFEVENKLEPDRKLQLANELGLQPRQIAVWFQNRRARCKTKHLETEYETLHSSYKSLKMDYDYLVKENEKLKAEVLHLKHDGTAQDVEHASSGESDTKELSEAIPVSTDEEDHKVSVSTSKNDEQSSTKSDVTNEDSPRLTDGIHHPLFVKSGESPHDFDPSQSVLSLHGEDCLNEEMLQRAYVFPKIEDAYCHPHATSCGYGFSVEDHAFNFWSY

>SmHD-Zip8

MESGRIFFEPSCQNNMLFLGNADSGFRVGGRTVINMEESSKRRPFFSSAEELYDEDYFDEQLPEKKRRLTPEQVHMLEKSFEAENKLEPERKTQLAKKLNLQPRQVAVWFQNRRARWKTKQLERDYDQLKSSYDSLLCNYDSILKENDKLKSELLSLTEKLQPEETAGELSQKPDPVAAADTVQPCLELTVKVEDRLSTGSDGSAVVDDDGRQLLDSGDSYFPDNDYPDCMAAVDGINSEEDDGSDDGRSYFTDVFAVAEQQEPIGWCIWS

>SmHD-Zip9

MDWNGNIKMPYVSRPVDTSNFSSLYNCSFDHFPGLEMKQQTMLPMIMNKNMNMNMNMNNLGVDPKKKRLSNEQLELLENSFHEEIKLEPDRKMKLAKELGLQPRQIAIWFQNRRARWKGKRLEHLYDALKQEYDAVARDKHKLQQEVIALRAILKDQVAKKLVLSTGYTDISGEETVESTSIPSSDNHPPAAAAPVAGLHQIEECNYTLNMADYNNTMAATPPYWATSMPSCP

>SmHD-Zip10

MYKNSKAHGVDKLTCGLASGLDLSTSLARTHTRAPPPTIDRSIDRTDDDVAAGRAGRVGSPELAGLWCYIQRDEVVLPLGKIRLTEEHYRGDNLLAGRDSSRLDLKIIFSAVDTRSRNGSHRRQYGWLHHLCNHRFNHVVNIFLVCINGASSSTSRTIGLAEKPSTFSGKNFKYWQEKMLFYLTTVGFAGFLMEDKPPTPSEGEGETSQEIRAGYLNWCRGDYLCRNTVLMSLDERLYRVFKHIMDWNGNLRMPFVSRPADTSNFNFLYNCATFDHFPEMKQQSETMMEKSMNNLGVDPKKKRLSNEQLELLEISFQEEIKLDPDRKMKLAKELGLQPRQIAIWFQNRRARWKGKQLERLYDALKQEYDAVSRDKQKLQQEVMALRAILKDQVGKNQVSTGYTDISGEETVESTSIPSSDPTATATAAEEAMTAAASLHQMAECNYPMNMEDYNNTVMIAPYWATSMPSCP

>SmHD-Zip11

MKRLSSSDSLGGLMSICPNSTVDVDEHNHGGNPMYTREFQTMLEGLEEEESGQISEKKRRLSVDQVKALEKNFEVENKLEPERKVKLAQELGLQPRQVAVWFQNRRARWKTKQLERDYGLLKANHEALKHKYESLHRENQSLLNQIRELNGKIGVKEEASVSESEEKCDVEIDEGGGAMEHQENEMMGAVFGDFKDGSSDSDSSAILNEDNNSPITSPAGSSTTTALTADSRRPRRVLL

>SmHD-Zip12

MSTSIKKGTKNSRRKRFSDDQIKSLETMFETEARPELHLKQHLANKLGLQPRQIAIWFQNKRARSKSKQIEQEYSVLKSNYDNLALQFEALRKENQTLLVQVQKLRKMADNKDCEENDKNQIKLAASEIPRLLLDISGDELCMPLCSELNGREDYLEEEADVLNMAQIAESSLASPENGCSLESCTFLDNTGGTAQWWDF

>SmHD-Zip13

MKRLSNSDSLGGLMSICPNATEEESNNPMYAREFQTMLEGLEEEESGHVSEKKRRLSVDQVKALEKNFEVENKLEPERKVKLAQELGLQPRQVAVWFQNRRARWKTKQLERDYGGLKASYDGLKLNFERLQRDNESLLKELNGDDEEKTRLESEIGGAEAEMENPEGGKKEEASWPKFGDGSSDSDSSAILNEENIGFQLGAPQLVKIEEHNFFGEESCSTLFSDDQPPSLPWYCNDQWD

>SmHD-Zip14

MSXPRLKNIALPRHCSPSADAAAPKSTRAAVQPHVSEKKRRLSVDQVKALEKNFEVENKLEPERKVKLAQELGLQPRQVAVWFQNRRARWKTKQLERDYSGLKASYDGLKLNFERLQRDNESLLKEIRELKSKLNGDDEEKTRLESEIGDAVLRRAGAAEERGRGRGLPRAEGEHKLADVDVGEVEEEDVHVRAGEIQARDGAGRYQAEAGQAGGEAHCPGEGRALAARARAGKGSDAD

>SmHD-Zip15

MIYSGMEMKQAMVAEKSNSNSSMSINYGGGDPKKKRLSTEQLESLENSFQEEIKLDPDRKMKLAKELGLQPRQIAVWFQNRRARWKGKQLERLYDALKQEFDAVSREKHKLHQEVLALRAILKDQVAKKQVSTDVSGEETVESTSIPSSEKGRGEGNNQMGECNYVLNVEDYSPLIMTPYNWGALP

>SmHD-Zip16

MMASSPQKTPPKNKTKTHHHHHHHPRRFSEDQIKSLESIFEQESKLEPKKKLEIARDLSLHPRQVAIWFQNKRARWKSKRIEQEFIHLKSTYDALSISFDALKREKHSLQMQLQELNDRLKNRGDRINDDDEDERIITSCFDGQEFLKRGEEVMSQTWRNLSDSGVLFHDSSENPNWWET

>SmHD-Zip17

MDWNGNIKMPYVSRPVDTSNFSSLYNCSFDHFPGLEMKQQTMLPMIMNKNMNMNMNMNNLGVDPKKKRLSNEQLELLENSFHEEIKLEPDRKMKLAKELGLQPRQIAIWFQNRRARWKGKRLEHLYDALKQEYDAVARDKHKLQQEVIALRAILKDQVDKKLVLSTGYTDILGEEQLKAHPFQAPTTTLPRRQRRWRAFTRLRNATTH

>SmHD-Zip18

MYKDGNCKLIINIVGVASLLAKRPQTMAFSSGIEEEMHNNNNGGGSVGDDDFSDDGSQIGEKKRRLNMEQVKTLEKNFELGNKLEPERKMQLARALGLQPRQIAIWFQNRRARWKTKQLEKDYEILKRQFEAVKAENDALHSHNHKLHAQIVALKNREPTESINLNKETEGSCSNRSENSSENVKLDISRTQAIDSPISGRQLFPPSLRPAAAQQLFHNSKMEHAVKEESLCNMFVGMDDQTGFWPWLEQQQFN

>SmHD-Zip19

MAGSAAVYGGGDAPNQAKKRNFDQPLDSIAAESSLGSNTMLNFGAVGGVKRSRNMLFSSLDQEEFGEEYLDEYFNQQEKKRRLTADQVQFLERSFEADNKLEPERKVQLANELGLKPRQIAVWFQNRRARWKTKQLETDYETFHARYETLKSDYDNLVKENDKLKAEVLRLKQNDVGGDTEKVNLHLLDNKGLPKAMPREANARVISEDEESNGSVFAFKNEEQSSAKSEASNVGSPRLTDGIHSPLLESGITSHAFDRDYSDSSQIGEDNLHEEPDYVFPLSSFYYDGFPVEDHAFNFW

>SmHD-Zip20

MGFDDLCNTGLVLGLGFSSSASKKPSKNLEPSLTLSLSGETYDLAVKKADSDNNLYRQDSAASSFSNVSVKREREMGSEEVEIERRVSDEDDDGPNGRKKLRLTKTQSALLEESFKQHSTLNPKQKQDLARELKLRPRQVEVWFQNRRARTKLKQTEVDCEFLKKCCETLTDENRRLQKELQELKALKLAQPLYMQLPAATLTMCPSCERIGGAAADNTAKTSYVTKPHFYNPFTNPSAAC

>SmHD-Zip21

MEILNPNSTCLELSIAMPGFSSSPSSSEAKGEMKLLDMNEAPSRSEEECVEEEDEDCPYGGGGPIPRKKLRLTKDQSRRLEQSFLQNQALNPKEKEALAVELRLKPRQVEVWFQNRRARSKMKQTEIECEYLKRWLGALTEQNRKLQKEVEELRFIKAAPPPRQVSALTMCPRCERTTLLDKGRATTAAASEGKVPFLRHSSDPLVIYK

>SmHD-Zip22

MENCHSAPFCLELTIGFSSSSFLSPGESTMKELDINQVPSSGTEEECMEEEEEGESPNGGPARRKKLRLTKEQSRLLEESFKQNHTLNPREKETLAEILSLKPRQVEVWFQNRRARSKLKQTEMECEYLKRWFGSLSEQNRKLHKEVEELRAINVHGHGHLPPPASALSMCPRCERVTAAANTKMSHLRHRQPPAACFL

>SmHD-Zip23

MSIKGWQWAGSGPDPAWSRSRSVFLCLDPDPVQIHWIQKNEIQTQIHEIHWISGPDPDPYCALAKQLNLRPRQVEVWFQNRRARTKLKQTEVDCEYLRRCCENLTEENRRLQKEVNELRALKLSPQLYMNMSPPTTLTMCPQCERVAVSSSSTLRLRVLCSAHVETGDFTLLCSAADRRWGRPALQQTGGGRRPEVGKAGGADTGGWVSRAIGAWRIGERRRWDGRSRVSNLKWNFKL

>SmHD-Zip24

MGVDDDSCQTRLVLGLGIRSSAENSKKSLSSSPSLTLTLFGETINKRVDVCSNDSAASSLSNASVKREREVGSEEVDETERVSSRGSDEDDDGSNGRKKLRLTKTQSALLEESFKHHSTLNPKQKQDLAMELKLRPRQVEVWFQNRRARTKLKQTEVECELLKKCCETLTDENRRLQKELHDLKLAPPLYMKLPAAATLTICPTCERFSSSGGGAAENATKSPFAKPHFYNHFTNPSAAC

>SmHD-Zip25

MKKMSRSDKRRFSDEQIRRLEVIFEAESKLEARKKAEVARELGLQPRQVAIWFQNRRARWKSKHIEKEYAALLANYNALAAEFDSLKKEKHSLLNQLEKLKGKGDDDEEGGSGARCGGESESVKTNDFEVKNEGMVSVSDEEEMLRMVETTSDWGGLDCDDIIIDQSSSYQWWGFWS

***Arabidopsis thaliana***

>ATHB1

MESNSFFFDPSASHGNSMFFLGNLNPVVQGGGARSMMNMEETSKRRPFFSSPEDLYDDDFYDDQLPEKKRRLTTEQVHLLEKSFETENKLEPERKTQLAKKLGLQPRQVAVWFQNRRARWKTKQLERDYDLLKSTYDQLLSNYDSIVMDNDKLRSEVTSLTEKLQGKQETANEPPGQVPEPNQLDPVYINAAAIKTEDRLSSGSVGSAVLDDDAPQLLDSCDSYFPSIVPIQDNSNASDHDNDRSCFADVFVPTTSPSHDHHGESLAFWGWP

>ATHB3

MYMYEEERNNINNNQEGLRLEMAFPQHGFMFQQLHEDNAHHLPSPTSLPSCPPHLFYGGGGNYMMNRSMSFTGVSDHHHLTQKSPTTTNNMNDQDQVGEEDNLSDDGSHMMLGEKKKRLNLEQVRALEKSFELGNKLEPERKMQLAKALGLQPRQIAIWFQNRRARWKTKQLERDYDSLKKQFDVLKSDNDSLLAHNKKLHAELVALKKHDRKESAKIKREFAEASWSNNGSTENNHNNNSSDANHVSMIKDLFPSSIRSATATTTSTHIDHQIVQDQDQGFCNMFNGIDETTSASYWAWPDQQQQHHNHHQFN

>ATHB5

MKRSRGSSDSLSGFLPIRHSTTDKQISPRPTTTGFLYSGAGDYSQMFDALEDDGSLEDLGGVGHASSTAAEKKRRLGVEQVKALEKNFEIDNKLEPERKVKLAQELGLQPRQVAIWFQNRRARWKTKQLERDYGVLKSNFDALKRNRDSLQRDNDSLLGQIKELKAKLNVEGVKGIEENGALKAVEANQSVMANNEVLELSHRSPSPPPHIPTDAPTSELAFEMFSIFPRTENFRDDPADSSDSSAVLNEEYSPNTVEAAGAVAATTVEMSTMGCFSQFVKMEEHEDLFSGEEACKLFADNEQWYCSDQWNS

>ATHB6

MMKRLSSSDSVGGLISLCPTTSTDEQSPRRYGGREFQSMLEGYEEEEEAIVEERGHVGLSEKKRRLSINQVKALEKNFELENKLEPERKVKLAQELGLQPRQVAVWFQNRRARWKTKQLEKDYGVLKTQYDSLRHNFDSLRRDNESLLQEISKLKTKLNGGGGEEEEEENNAAVTTESDISVKEEEVSLPEKITEAPSSPPQFLEHSDGLNYRSFTDLRDLLPLKAAASSFAAAAGSSDSSDSSALLNEESSSNVTVAAPVTVPGGNFFQFVKMEQTEDHEDFLSGEEACEFFSDEQPPSLHWYSTVDHWN

>ATHB7

MTEGGEYSPAMMSAEPFLTMKKMKKSNHNKNNQRRFSDEQIKSLEMMFESETRLEPRKKVQLARELGLQPRQVAIWFQNKRARWKSKQLETEYNILRQNYDNLASQFESLKKEKQALVSELQRLKEATQKKTQEEERQCSGDQAVVALSSTHHESENEENRRRKPEEVRPEMEMKDDKGHHGVMCDHHDYEDDDNGYSNNIKREYFGGFEEEPDHLMNIVEPADSCLTSSDDWRGFKSDTTTLLDQSSNNYPWRDFWS

>ATHB12

MEEGDFFNCCFSEISSGMTMNKKKMKKSNNQKRFSEEQIKSLELIFESETRLEPRKKVQVARELGLQPRQVAIWFQNKRARWKTKQLEKEYNTLRANYNNLASQFEIMKKEKQSLVSELQRLNEEMQRPKEEKHHECCGDQGLALSSSTESHNGKSEPEGRLDQGSVLCNDGDYNNNIKTEYFGFEEETDHELMNIVEKADDSCLTSSENWGGFNSDSLLDQSSSNYPNWWEFWS

>ATHB13

MSCNNGMSFFPSNFMIQTSYEDDHPHQSPSLAPLLPSCSLPQDLHGFASFLGKRSPMEGCCDLETGNNMNGEEDYSDDGSQMGEKKRRLNMEQVKTLEKNFELGNKLEPERKMQLARALGLQPRQIAIWFQNRRARWKTKQLEKDYDTLKRQFDTLKAENDLLQTHNQKLQAEIMGLKNREQTESINLNKETEGSCSNRSDNSSDNLRLDISTAPPSNDSTLTGGHPPPPQTVGRHFFPPSPATATTTTTTMQFFQNSSSGQSMVKEENSISNMFCAMDDHSGFWPWLDQQQYN

>ATHB16

MKRLSSSDSMCGLISTSTDEQSPRGYGSNYQSMLEGYDEDATLIEEYSGNHHHMGLSEKKRRLKVDQVKALEKNFELENKLEPERKTKLAQELGLQPRQVAVWFQNRRARWKTKQLEKDYGVLKGQYDSLRHNFDSLRRDNDSLLQEISKIKAKVNGEEDNNNNKAITEGVKEEEVHKTDSIPSSPLQFLEHSSGFNYRRSFTDLRDLLPNSTVVEAGSSDSCDSSAVLNDETSSDNGRLTPPVTVTGGSFLQFVKTEQTEDHEDFLSGEEACGFFSDEQPPSLHWYSASDHWT

>ATHB20

MYVFDPTTEAGLRLEMAFPQHGFMFQQLHEDNSQDQLPSCPPHLFNGGGNYMMNRSMSLMNVQEDHNQTLDEENLSDDGAHTMLGEKKKRLQLEQVKALEKSFELGNKLEPERKIQLAKALGMQPRQIAIWFQNRRARWKTRQLERDYDSLKKQFESLKSDNASLLAYNKKLLAEVMALKNKECNEGNIVKREAEASWSNNGSTENSSDINLEMPRETITTHVNTIKDLFPSSIRSSAHDDDHHQNHEIVQEESLCNMFNGIDETTPAGYWAWSDPNHNHHHHQFN

>ATHB21

MNNQNVDDHNLLLISQLYPNVYTPLVPQQGGEAKPTRRRKRKSKSVVVAEEGENEGNGWFRKRKLSDEQVRMLEISFEDDHKLESERKDRLASELGLDPRQVAVWFQNRRARWKNKRVEDEYTKLKNAYETTVVEKCRLDSEVIHLKEQLYEAEREIQRLAKRVEGTLSNSPISSSVTIEANHTTPFFGDYDIGFDGEADENLLYSPDYIDGLDWMSQFM

>ATHB22

MEYWSSSFIDGASSSSFISPFYNFDHFSGNQDNRCLGTMMGAQQDILHVPLAMVESGYGEESNSFNGQEKKKKKMTSEQLKFLERSFQEEIKLNPDRKMKLNPDRKMKLSKELGLQPRQIAVWFQNRKARWKNKQLEHLYESLRQEFDIVSREKELLQEELIQLKSMIREDSSCKKKQTWEKACS

>ATHB23

MSCNNNGLAFFPENFSLQNHHQEEEDHPQLLQDFHGFLGKRSPMNNVQGFCNLDMNGDEEYSDDGSKMGEKKRRLNMEQLKALEKDFELGNKLESDRKLELARALGLQPRQIAIWFQNRRARSKTKQLEKDYDMLKRQFESLRDENEVLQTQNQKLQAQVMALKSREPIESINLNKETEGSCSDRSENISGDIRPPEIDSQFALGHPPTTTTMQFFQNSSSEQRMVKEENSISNMFCGIDDQSGFWPWLDQQQYN

>ATHB40

MNYTVDDQNMAFISQLYPDVYTQIVQPVGEVKQPKRRRKKTKGSVASADGGNGLFRKRKLTDEQVNMLEMSFGDEHKLESERKDRLAAELGLDPRQVAVWFQNRRARWKNKRLEEEYNKLKNSHDNVVVDKCRLESEVIQLKEQLYDAEREIQRLAERVEGGSSNSPISSSVSVEANETPFFGDYKVGDDGDDYDHLFYPVPENSYIDEAEWMSLYI

>ATHB51

MEWSTTSNVENVRVAFMPPPWPESSSFNSLHSFNFDPYAAGNSYTPGDTQTGPVISVPESEKIMNAYRFPNNNNEMIKKKRLTSGQLASLERSFQEEIKLDSDRKVKLSRELGLQPRQIAVWFQNRRARWKAKQLEQLYDSLRQEYDVVSREKQMLHDEVKKLRALLRDQGLIKKQISAGTIKVSGEEDTVEISSVVVAHPRTENMNANQITGGNQVYGQYNNPMLVASSGWPSYP

>ATHB52

MENSQSQGKNKKKRLTQDQVRQLEKCFTMNKKLEPDLKLQLSNQLGLPQRQVAVWFQNKRARFKTQSLEVQHCTLQSKHEAALSDKAKLEHQVQFLQDELKRARNQLALFTNQDSPVDNSNLGSCDEDHDDQVVVFDELYACFVSNGHGSSSTSWV

>ATHB53

MDHGRLMDDQMMLGSQVYPYTTQPQNSHCIIVNQIDGGEESKPVKRRRKRRSKGSSATNEEDVAEIGGMLRKRKLTDEQVNMLEYSFGNEHKLESGRKEKIAGELGLDPRQVAVWFQNRRARWKNKKLEEEYAKLKNHHDNVVLGQCQLESQILKLTEQLSEAQSEIRKLSERLEEMPTNSSSSSLSVEANNAPTDFELAPETNYNIPFYMLDNNYLQSMEYWDGLYV

>ATHB54

MENSDTDSEVFFWFQNQNQNHSHKFPSSCFPPSSHSAFYGSSSMINTETATMDEEDVCESYMMREITKKRKLTPIQLRLLEESFEEEKRLEPDRKLWLAEKLGLQPSQVAVWFQNRRARYKTKQLEHDCDSLKASYAKLKTDWDILFVQNQTLKSKVQFLNRLTSHYFQESVQNFDDTFKQVDLLKEKLKMQENLETQSIERKRLGEEGSSVKSDNTQYSEEEGLENQYSFPELAVLGFYYDPTLTASNLRQEPLKVTCADQMTQIQISDVTEPASSAHKKIEVTQRSSSMSRKRDKPYTNRHTPARISKRRRPWAPSSSEHDEIIDKPITKPPPPPALVVMGLPANCSVLELKSRFEIYGSISRIRIHKDGIGSVSYRTAESAEAAIAGSHEPSFGISIDSKKLEVVWATDPLVKWKEGVTAGEGKERTSSFSSKLLRPVMPLRKHGRSSRLASAIVNPRSDNTKGISGDGGISSPATTSEVKQRNIVTYDDIV

***Nicotiana tabacum***

>NtHDZI1

MKRPCSSDSLGALMSMCPNTTDEQNHMYSTRDFQSMLLDEEGCIEESGHVSEKKRRLSVEQVKALEKNFEVENKLEPERKVKLAQELGLQPRQVAVWFQNRRARWKTKQLERDYGVLKANFDALKHNYESLKHDNEALLKEIRELKSKVYTENGENRGVAVKEEAMESESDDNKVIELSKPNDNDINNNNLLENFEEEEDEEEEINFENFNAAAASTNIFGDHFKDGSSDSDSSAILNEDSSPNAAAISSSGAFLISTNGNGSGSSKSSTSLNFCFQFTESSSKSNLGDGQKGNNYYQPQQYVKMEEHNFFNGEESCSTLFTDEQAPTLQWYCPEDWNWKES

>NtHDZI2

MKRPCSSDSLGALMSMCPNTTDEQNHVYSTRDFQSMLLDEEGCIEESGHISEKKRRLSVEQVKALEKNFEVENKLEPERKVKLAQELGLQPRQVAVWFQNRRARWKTKQLERDYGVLKSNFDALKHNYESLKHDNEALLKEILELKSKVYTENGESKGVAVKEEAMESESDDNKVIEQSKPNDNDNNNNNFLENFEEDDEEEEINFENFNVAAAATSTNIFGDNFKDGSSDSDSSAILNEDNSPNAAAISSSGAFLISTNGNGNGNGSSTSLNFCFQFTESSSKSNLGDGQKGNNNYYQPQQYVKMEEHNFFNGEESCSTLFTDEQAPTLQWYCPEDWNWKE

>NtHDZI3

MGSEVEELIKKEKYGVVPNGDNEDLYNFPKFTKICVILVGFGDDHNQWNSQVYSRNFQSMLELGLEDEPCAEESGHGSEKKRRLRVDQVKALEKNFEVDNKLEPERKVKLAQELGLQPRQVAIWFQNRRARWKTKQLERDYNVIKANFDSLKHNYESLQHDNEALLKEICDLKSKLNGGNNESKADVKEEAIESETDDKDIDQSKPNNSTTSLGNCEDDSTELNIESFNGGNGIITSSNIFADFKDGSSDSDSSAILNEENSPNYAAISSSGAFLISNNGGSNSSSSNSSLNCFQFFDSNPKAILGDGQDKVVYNNQPQFVKMEEHNFFSSEESCSTLFADEQPPTLQWYCSEDWNNWKDS

>NtHDZI4

MLELGLDEEACVEESGHGSEKKRRLRVDQVKALEKNFEVENKLDPERKVKLAQELGLQPRQVAIWFQNRRARWKTKQLERDYNVLKANFDSLKHNYESLQHDNEALLKEICELKSKQNGGNNESKADVKEEAIESETDDKYIDQSKPNNSTTLGNCEDDSTELNFESFNGGNGTINDSNIFADFKDGSSDSDSSAILNEENSPNYAAISSSGAFLISNNGGSNSSSSNSSLNCFQFFDSNPKAILGDAKDKVFCNNQPQFVKMEEHNFFSSEESCSTLFADEQAPTLHWYCPEDWNNWKDS

>NtHDZI5

MKRFSFSDSSGKLLYPSEEKNTKNLAYSTDFQAMLDGLEDEDGIEENGCVTGKKRRLKVDQVQALEKIFEVDNKLDPERKVKIAQELGLQPRQVAIWFQNRRARWKTKQLERDYNILKANYEALQLNYTKVEQEKEGLINELKGLKEKIGEENSTAFQSPQEILELNPRNYEVNNKLYSDFVDSKDGSSDSDSSGVMNEENYNNTLNYQPLMPKVSSYNALDHLSLSSSTYTQLLDPRASSTSSMRGYNQQQQIGRMEEQNGFTTEDSCNIYSVDQAPNLYWYLSDHRN

>NtHDZI6

MKRFSFSDSSGKLLYPPEEKNTENLAYSTDFQAMLDGLEDEDGIEESGCVTGKKRRLRVDQVQALEKIFEVDNKLDPERKVKIAQELGLQPRQVAIWFQNRRARWKTKQLERDYNILKANYEALQLNYTKVEQEKEGLINQLKGLKEKIGEENSTAFQRPQEILELNPRNYEVSNKLNSDFVDSKDGSSDSDSSGVMNEENYNNTLNYQPLMPKVSSYNALDHLSLSSSTYTQLLDPRASSTSSMRGYNQQQQQVGKMEEQNGFTTEDSCNIYSVDQAPNLYWYLSDHRN

>NtHDZI7

MLFLGNSDPVFRGPRSMMKMEDSSKRRPFFSSPEELYDEEYYDEQSPEKKRRLTPEQVHLLEKSFEAENKLEPERKTQLAKKLGLQPRQVAVWFQNRRARWKTKTLERDYDQLKSSYDSLLSDFDSIRKDNEKLKAEVVSLMEKLQGKVVGEAGVINEKCEVLEVDALTLQVKVKAEDRLSSGSGGSAVVDEHSPQLVDSGDSYFHTGHMEEYPLGAGGCNNIPPPMDGLQSEEDDGSDDGGGHGYFCNVFVAAEEQQHEEGEPIGWFWS

>NtHDZI8

MEVGRVKEGSNMSTANSVLPSEVLHNLWMSNSSPSFHGPTSMVNFEDGRGEISKEMSFFSPIDKEETSTDDYDSCFHRPEKKRRLLPNQVQFLEKSFEVDNKLEPERKVQLAQELGLQPRQIAIWFQNRRARYKTKLLEKDYDVLKASFDKLKDDYDSLFKENENLRNEVDLLKEKMLRREKGKENSGQNEPISPVDAEEAQKATPNVVTSEVPSIQKVVCKQEDASSAKSDVIDSDSPHYTDGNHSSNAFEAEPSDFSQDEDDNLSKSFLCFPEIGDQIQANSCNLGFQIEDHQPCWFWQY

>NtHDZI9

MLFLGNSDPVFRGPRSMMKMEDSSKRRPFFSSPEELYDEEYYDEQSPEKKRRLTPEQVHLLEKSFETENKLEPERKTQLAKKLGLQPRQVAVWFQNRRARWKTKTLERDYDQLKSSYDSLLSDFDSMRKDNEKLKAEVVSLMEKLQGKVVGEAGVVGEKCEVLEVDAMTLQVKVKAEDRLSSGSGGSAVVDEHSPQLVDSGDSYFHTGHIEEYPIGAGGCNNIPPPMDGLPSEEDDGSDDGGGHGYFSNVFVAAEEQQHEDGEPIGWFWS

>NtHDZI10

MVNFDDTRGTKTKERSFFPSLDKEENSNEDYEVSFHQPEKKRRLLPKQVQFLEKSFEVENKLEPERKIQLAKETGLQPRQVAIWFQNRRARYKSKQLEKDYDVLKASFDKLKDEYDSLFKENDNLRNEVHLLKEKLFNREQNSEEKEPISPLNTEAQNPTNNMQNLTMMVCKQEDASSAKSDILDSDSPCYADGNYTSFLEPSDSSHAFETETSDFSQEDDSLSRTLLSSLCFPKLEDDLPVNSCHLSFQIEDQSWFCHY

>NtHDZI11

MLSGITGIMAFVPSTPEISINFQDNHIPSTSPKVFPSPCNIPHQDFNGVSSMLMRRSMSFSGVERCDNHQDLRVDDNEMSDDDGSSQLLGEKKRRLNLEQVKALERSFEVANKLEPERKIQLARALGLQPRQIAIWFQNRRARWKTKQLERDYEILKRQYDALKSDNDALKTQNKNLHSELQLLTLRNRESGGGGAPMINLNKENEGSWSNGSEENNSIDVNIGTTTTTRTSSGDSPFYSNNNKNNVFPESSPIPLAQLFHQSSSRSDLQCHTIDPTVPDDQGFCNMFATPVNDQTNFWPWPEQQQFN

>NtHDZI12

MLSGTTGIMAFVPSTPEISINFQDNHIPSTSPKVFPSPCNIPHQDFNGISSMLMRRSMSFSGVERCDNHQDLRVDDSEMSDDDGSSQLLGEKKRRLNLEQVKALERSFEVANKLEPERKVQLARALGLQPRQIAIWFQNRRARWKTKQLERDYEILKRQYDVLKFDNDALKTQNKNLHSELQALTLRNRESGGGGAPMINLNKENEGSWSNGSEENNSIDVNIGTTTTTRTSSGDSPFYSNNNKNNVFPESSPIPLAQLFHQSSSRSDLQCHKIDPTVPDDQGFCNMFATPVDDQTNFWPWPEQQQFN

>NtHDZI13

MAPGMLYSAGAGACASNFDGVLIQKQRVMSSSSSTAKEHLGSLFVPGCSSSYLGSSSMVSFGSVNGGKRSFFDSFDQEENEADELGEYFHQAEKKRRLTENQVQFLEKSFGEENKLEPERKVQLAKELGLQPRQIAIWFQNRRARWKTKQLEKEYEALRNQYDNLKSDYNNLLKEKENLRAEVFQLNDKLLLNEKENGQLTQRDFQKHSDSSPKETMADPISRVKMSNVSARVLKEDLSSAKSDVLGSESPHYTDGVHSSFVKQGDSYVVEPEPSDLSQDDEENFNKTMLSTANLLAKAEDDDYRVTSSNVSYFGFPVEDQGFGFWSY

>NtHDZI14

MTCTNEMDFFPANFMLQTSHEDEHQPSTSLTPILPSCSPQDFHGIASFLGKRSMSFSGMEAINNNNCEENHNGEEDLSDDGSQAGEKKRRLNMEQVKTLEKNFELGNKLEPERKMQLARALGLQPRQIAIWFQNRRARWKTKQLEKDYEILKRQFEAIKAENDALQSQNQKLHAEVMALKNRDQPTESINLNKETEGSCSNRSENSSEIKLDISRTPAIDSPLSTHHPITSRPFFPPSIRPNNNGVAQLFNMNPNTISRPDLKLMDQNSTVKEESSLSNMFCGIDDQTAFWPWLEQQHFN

>NtHDZI15

MTCTNEMDFFPANFMLQTSHEDEHQPSTSLTPILPSCSPQDFHGIASFLGKRSMSFSGMEANNNNCEENHNGEDDLSDDGSQAGEKKRRLNMEQVKTLEKNFELGNKLEPERKMQLARALGLQPRQIAIWFQNRRARWKTKQLEKDYEVLKRQFEAIKAENDALQSQNQKLHAEIMALKNRDQPTESINLNKDTEGSCSNRSENSSEIKLDISRTPAIDSPLSNHPITSRPFFPPSIRPNNNGVAQLFNMNPNSISRPDLKLMDQSSTVKEESSLSNMFCGIDDQTAFWPWLEQQHFN

>NtHDZI16

MTTTNTVLHSEVLHNLWMSNSSPSFHGPTSMVNFEDGGGEISKERSFFSPIDKEETSNDDYDSCFHRPEKKRRLLPNQVQFLEKSFEVDNKLEPERKVQLAQELGLQPRQIAIWFQNRRARYKTKLLEKDYDVLKASFDKLKDDYDTLFKENENLRNEVDLLKEKLLEREKGKENSEQSEPINPVDAEAQKATPIVVTSEVPSIQKVECKQEDASSAKSDVIDSDSPHYTDGNHSSNAFEPEPSDFSQDEDDNLSKSFLCFPEIGDQIQANSCNLGFQIEDQPCWFWQY

>NtHDZI17

MAPGMLYSGGSGAFNFDGVLIQKQRVMSSSSTTTAKEHLGSLFVPGSSSSSYLGSSSMVSFGRVNGGKRSFFDSFDQEENEADELGEYFHQAEKKRRLTENQVQFLEKSFGEESKLEPERKVQLAKELGLQPRQIAIWFQNRRARWKTKQLEKEYEALRNQYDNLKSDYNNLLKEKENLRAEVFQLTDKLLLKERKNGQLTLPDFQKHSDASPKETMADPISRVKMSNVSAQVLKQQQEDLSSGKSDILDSESPHYTDGVHSSFIEQGDSSYVFEPEPPDLSQDDEEKFNKTMLSTANLLAKAEDDDYRVTSSNVSYFGFPVEDQGFGFWSY

>NtHDZI18

MEVARVYEGSNVNSHNGFILPNEIFPPPSKVLDSLWISNSSPTFHGSVSMVNFNDTRGKKTKERSFFPSLDKEENSNEDYQVSFHQPEKKSRLLPKQVQFLAKSFEVENKLEPERKIQLAKEIGLQPRQVAIWFQNRRARYKSKQLEKDYDVLKASFDKLKDEYDSLFKENENLRNEVHLLKEKLFDRVNGEQNSEQKEPISPLNTEAQNSTTNMQNLTMMVCKQEDASSAKSDILDSDSPCYADENYTSFLEPTDSSHVFETETSNFSQEDDSLCWSLLPSLCFPKLEDDLPVNSCNLSFQIEDQSWFCHY

>NtHDZI19

MTCTNEMAFFPANFMLHTPHQDDHQDFQGVGSFLGKRTMSFNGMEANNNCEENHEEEDLSDDGLREAGRKKKIRRLNMEQVKTLEKNFELGNKLEPERKMQLARALGLQPRQIAIWFQNRRARWKTKQLEKDYDVLKRQFEAVKAENDSLLAHNQKLHAEIMTLKNVEQPTEISINLNKETEGSSSNRSENSSEIMKLDTSMRPAAIDSPILVSTTHQPITRCSRLLFPSPMRPNGVAQLFHSSRQEVLHCNMKLMDQTVKEETLSNMLSAIDDQSPGFWPWLEQPHFN

>NtHDZI20

MSFSGMEANNNCEENHEEEDLCDDGLHEAAREKKIRRLNMEQVKTLEKNFELGNKLEPERKMQLARALGLQPKQIAIWFQNRRARWKTKQLEKDYDVLKRQLEAVKAENDSLLAHNQKLHAKIMALKSGEQPTELSINLNKETEGSSSENSSEKMKLDSTMTAAAIDGPLILVSTAHQPITRGSRLLFPPPVRPNGVAQLFQRQQVLQSDMKLMDQTVKQETLSNMFVDDQSPGFWPWLEQPHFN

>NtHDZI21

MAGPELFYGDAAGFNGILLQNQGLMSSFATKEAVGSPASFLASGSMVSFRGVNGGNKSARSFFQSVEENETDELEEYLHQPEQKRRLTADQLQFLEKSFEVENKLEPERKDQLAKELGLQPRQIAIWFQNRRARWRTKQLENDYEALRNKYDNLKADYINLLKEKDDLRAQVIQFTDKLLLKEKEKGELATSDIKELSDASPKENMIDQKVSAITLKQHEDLNSVKSDAFDSESLVQSSILDQSDLSQDEEENLSKNLVVHDHYPMTSSNLSCFGYPGEDQGFGFWSF

>NtHDZI22

MLFSSTMAFVPSTAQETSSVNFRNHEAANDDLPSSFPSHVFPSSCAPQQEFQGISPLLMRRAMSFDHNQESRVDDDMSDDDGSSHLLGEKKRRLNLEQVKALERSFEMGNKLEPERKMQLARALGLKPRQIAIWFQNRRARCKTKQLEKDYEILKRQCDKLKIDNDALKTQNKKLHSELLSLGNRESAGDTPPILFNLNKENEGCNWNINGSGDENNSTIDVNLGTTTTRTSSTNSPLNSHDIFPTAYKVEQTVNEEGFCNMFTNVEDQTNFWPLPVQQHFY

>NtHDZI23

MTNLYAGISPLLMRRAMSFDHNQESRVEDDMSDDDGSSHLLGEKKRRLNLEQVKALERSFEMGNKLEPERKMQLARALGLKPRQIAIWFQNRRARCKTKQLEKDYEILKRQCDKLKADNDALKTQNKKLHSELQLLSLGNRESAGGTPPMLFNLNKENEGCNWNNNGSGDENNSTIDVNLGTTTRTSSTNSPLNNHDVFPTAYKVDQTVNEEGFCNMFTNVEDQTNFWPLPVQQHFY

>NtHDZI24

MDWNGNLRPFVSRPAPPDNSFNLLYSYNYDQYPGMEMKHAMQTQQGGVLPTMDKLNNNFAMNQLDKKKRLTSDQLESLENSFQEEIKLDPDRKMKLAKELGLQPRQIAVWFQNRRARWKSKQLERLYDSLKQDYDLVSREKQKLQNEVLALRAILKEQATKKQVNSTVYTEISGEETVESTSMPSSNKTRGITTSNHQNNNMAECSYVFNVNNVEGFNPVMPPYWV

>NtHDZI25

MDWNGNLRSFVSRPAPPDNSFNFLYSYNYDQYPGMEMKHAMQTPHSGVLPTMDKLNDNFAMNQLDKKKRLTSDQLESLENSFQEEIKLDPERKIKLAKELGLQPRQIAVWFQNRRARWKSKQLERLYDSLKQDYDLVSREKQKLQNEVLALRAILKEQATKKQVNSTVYTEISGEETVESTSMPSSNKTRGISTSNHQNNNMAECSYVFNVNNVEGFNPVMPPYWV

>NtHDZI26

MNHQVAADEMVLISQYYPDIYTQLVPQQGESKPRRRRKKKQGEGSGSGVMRKRKLSDEQVNLLEQSFGNEHKLESERKDKLASELGLDPRQVAVWFQNRRARWKSKKLEEEYSKLKSDHDTNVVEKCRLESEVLKLKDQLSEAEKEIQRLLLERCDGVSSNNSPSTSSFSMEAAMEPPFLGEFGMEGLDNVFYTPDNTYVQGLDWVNLYI

>NtHDZI27

MIMSSSMNLQVDDQMVLISQYYPGIYTQELPEQGEAKPRRRRKKSKGESSGIMRKRKLSEEQVNLLEQSFGSEHKLESERKDKLASELGLDPRQVAVWFQNRRARWKSKKLEEEYSKLKTEHDTNIVDKCRLENEILKLKEQLSEAEKEIQRLLMERCDGLNSSNSPTTSSLSMEAANMEPPFLGDFGMEGFDNTFYVPENTYSQGLEWVNLYMPYDM

>NtHDZI28

MNHQVAADEMVLISQYYPDIYTQLVPQQGESKPRRRRKKKQGEGSGSGIMRKRKLSDEQVNLLEQSFGSEHKLESERKDKLASELGLDPRQVAVWFQNRRARWKSKKLEEEYSKLKSDHDTNVVEKCRLESEVLKLKDQLSEAEKEIQRLLLERCDGVSSNTSPSTSSFSMEAAMEPPFLGEFGMEGLDNVFYTPDNTYVQGLDWVNLYI

>NtHDZI29

MNLQVDDQMILISQYYPGIYTQELPEQGEAKQRRRRKKNKGEASEVMRKRKLSEEQVNLLEQSFGKEHKLESERKDKLASELGLDPRQVAVWFQNRRARWKNKKLEEEYSKLKSEHDTNIVHNCRLENEVLKLKEQLSEAEKEIQRLLMERCDGVNSSNSPTTSSLSMEAANMEPPFLGEFGMEGFDNTFYVPENTYSQGLEWVNLYMPYDV

>NtHDZI30

MTHQLDDQMVLISQYYPDIYNQLVSEPGEAKPRRRRKKNKVEGSSSGMIMRKRKLSEEQVNLLEQSFEEEHKLETERKDKLASELGLDPHQVAVWFQNRRTRYKNKKLEEEYSKLKTEHDTAIVEKCRLENEVIKMKEQLSEAENEIQKLLLERNCDGVSSNSPISTSSFTMDQPPLLGEYGMEGILMDNLFFVAENTYNYHEGLEWVNPIYI

>NtHDZI31

MIHQLDDQMVLISQYYPDIYTQLVSEPGEAKQQRRRKKSKGEGSRSGVMRKRKLSEEQVNLLEQSFGNEHKLETERKDKLASELGLDPHQVAVWFQNRRARFKNKKLEQEYSKLKTQYDIAIVEKCRLENEVIKMKEQLSEAENEIQKLLLERNCDGVSSNSPISTSSFTMDQPPFLGEFGMEGMLMDNLFFIAENTYNYQEGLEWVNPVYI

>NtHDZI32

MFDGGEFSCTSSAAALNSAECFSSSSFSSLPSSKKKKVNNKNTRRFSDEQIKSLETMFENETKLEPRKKLQLARELGLQPRQVAIWFQNKRARWKSKQLERDYNILKSNFDNLASQCNSLKKENQSLLLQLQKLNDLMQKERGQYCSIGFDQESYNRDDNTIKNKEMEGKPSLSFDLSEHGVNGVISDDDSSIKADYFGLDEESDHLLKMVEAGDSSLTSPENWGSLEDDGLLDQQPNSSNYDQWWDFWS

>NtHDZI33

MEAEDTLTHGNTEPSNKKSKDGKRFTDEQVKLLESMFKQEIKLEPRKKLELARDLGLQPRQVAIWFQNRRARWKSKQMEHEYRLLKAEFDKLNMQFQSLKQEKESLLKQLEELNDQLENNHAGCSRGQDSLDSKIYTCSENGGTDLELKDNISGCLNASVEGKKVNEVENDREGRLIEHFRWKEEPEFWNMEELGDSSLGSPGHWCAVGLGRTFDLSCGNSKWWEF

>NtHDZI34

MEAEDTLTQGNTEPSNKKSKDGKRFTDEQVKLLEFMFKLETKLEPRKKLELARDLGLQPRQVAIWFQNRRARWKSKQMEHEYRVLKAEFDGLNMQFESLKQEKESLLKQLEELNDQLENNHAGCSRSQDSLDSEIYTSSENGGTDLELKDNIIPGCLNASLEDKRVEEVENDREGTLIEHFRWKEEPEFWNMEELGDSSLGSPGRWCAVGLGRTFDLSWEF

>NtHDZI35

MEPEESQELEKSKSPNSLKKNTENGKRFTDEQVKLLESMFRLETKLEPRKKLQLARDLGLQPRQVAIWFQNKRARWKSKQLENEYIVLKAKFDHLNMQFQSLKTEKEKLLIQLEILNDQLENNLARGSRSEDLKDNEMYTSSENGGTDLELKDSPGCSNARFDHKRIKEDQDDDTAEETDKYFTPKEEAEFWNLEELGDNSLEQWCVGLGSSFNQSCGNSKLWDF

>NtHDZI36

MSVGERHPTNSNMWSQSSLLGKDLNLHSMSSIKNCKDNMRRRFNDEQIRSLEHMFETEARPELRTKQQLAKSLGLQPRQVAIWFQNKRARSKSKQLEMEYRMLKISYDNLGSKYEMLKKENESLLIQLQRLRKLTEKGGNEEIQNEGNKLETEVIKSEFIPETCSPEFGLPLGEDSREIDYLGPESDILNMAQIADGLSEIENECNFESRTFLDDSDCRSPLWEF

>NtHDZI37

MSVGERPPTNSNMWSQSSLLGKDLNLHSMSSIKNCKDNMRRRFNDEQIRSLEHMFETEARPELRTKQQLAKSLGLQPRQVAIWFQNKRARSKSKQLEMEYRMLKISYDNLGSKYEMLKKENEALLIQLQRLRKITEKVGNGEIQNEGNKLETEVIKSEFIPETCSPEFGLPLCEDSREIDYLGTESDILNMAQIAYGLSEIENECNIESRIFFDDSDCRSPLWEF

>NtHDZI38

MDFFNSQTRKNNLKCHKKRLTQDQVRLLEISFNSNNKLDSDRKSQLAQELGLPPRQVAIWYQNKRARWKSQSLEVDYKALQQRLDNAMSDNERLKLEVERLKEELNKAQEMLLAFNTTTNNYSSISSSCDEVGSSCLQLHTQSKHHLDKELYACLIGDEGHFGHNFFASSMS

>NtHDZI39

MDFFNSQTRKNNLKCHKKRLTQDQVRLLEISFNSNNKLDSNRKSQLAQELGLPPRQVAIWYQNKRARWKSQSLEVDYKALQQRLDNAMLDNERLKLEVERLKEELNKAQEMLLAFNTTTNNYSSISSSCDEVGSSCLQLHDQSKHHLDKELYACLIGDEGHFGHNFFASSMS

***Oryza sativus***

>Oshox4

MKRPGGAGGGGGSPSLVTMANSSDDGYGGVGMEAEGDVEEEMMACGGGGEKKRRLSVEQVRALERSFEVENKLEPERKARLARDLGLQPRQVAVWFQNRRARWKTKQLERDYAALRHSYDSLRLDHDALRRDKDALLAEIKELKAKLGDEEAAASFTSVKEEPAASDGPPAAGFGSSDSDSSAVLNDVDAAGAAPAATDALAPEACTFLGAPPAAGAGAGAAAAASHEEVFFHGNFLKVEEDETGFLDDDEPCGGFFADDQPPPLSSWWAEPTEHWN

>Oshox5

MDPGRVVFDSGVARRACPGGAQMLLFGGGGSANSGGFFRGVPAAVLGMDESRSSSSAAGAGAKRPFFTTHEELLEEEYYDEQAPEKKRRLTAEQVQMLERSFEEENKLEPERKTELARRLGMAPRQVAVWFQNRRARWKTKQLEHDFDRLKAAYDALAADHHALLSDNDRLRAQVISLTEKLQDKETSPSSATITTAAQEVDQPDEHTEAASTTGFATVDGALAAPPPGHQQPPHKDDLVSSGGTNDDGDGGAAVVVFDVTEGANDRLSCESAYFADAAEAYERDCAGHYALSSEEEDGGAVSDEGCSFDLPDAAAAAAAMFGAAGVVHHDAADDEEAQLGSWTAWFWS

>Oshox6

MDGEEDSEWMMMDVGGKGGKGGGGGGAADRKKRFSEEQIKSLESMFATQTKLEPRQKLQLARELGLQPRQVAIWFQNKRARWKSKQLEREYSALRDDYDALLCSYESLKKEKLALIKQLEKLAEMLQEPRGKYGDNAGDDARSGGVAGMKKEEFVGAGGAATLYSSAEGGGTTTTTTAKLMPHFGSDDVDAGLFLRPSSQHHPPPPHAGAGFTSSEPAADHQSFNFHSSWPSSTEQTCSSTPWWEFESE

>Oshox8

MKRPSCRGSSMAIIHDTSDQQEDNMRSYMDGGGAAAYEEEEEEVEDDDGGGGGGGGGGGGGLGEKKRRLAAEQVRALERSFEADNKLDPERKARIARDLRLHPRQVAVWFQNRRARWKTKQIERDFAALRSRHDALRLECDALRRDKDALAAEIADLRDRVDGQMSVKLEAVAADEHQPPPPPPPPPLAYNSKVVDGSTDSDSSAVFNEEASPYSGAAIDHHHHQTPASYDTAGFTSFFAPSTTLTSSLSFPSMFHASSHFDGHQELLVGGGGAGAVADADLGGAGFFAGDEHAGGLSWYGAEGW

>Oshox12

MSREEDEKLLFPSFAFPAECFPEAATSGGEQKKARQRRRRKVKPEAAAALAGESGGDEQAKKRRLSDEQARFLEMSFKKERKLETPRKVQLAAELGLDAKQVAVWFQNRRARHKSKLMEEEFAKLRSAHDAVVLQNCHLETELLKLKERLADVEEEKAKLAAVAAATTGGGGGGGGGSSSPTSSSFSTVTYHPALAGQFGVEAAAEEADLTYMSEYAYNSYMLELAAAGYCGGVYDQFS

>Oshox13

MKRPTSSSRKSKKQGEDLAFSEEGSLPAVTMEQKDEAEMEEVDEEEEEEVDEDMAGGHAAQSPSPSCGLGEKKRRLALEQVRALERSFDTDNKLDPDRKARIARDLGLQPRQVAVWFQNRRARWKTKQLERDFAALRARHDALRADCDALRRDKDALAAEIRELREKLPTKPADTAASVKVEAGNDAAAGAAAATVCKDGSSDDSDSSVVFNDEASPYSGAAFIGFGPSFLVDDASAATVGCSSSLPALESKWHGPYSDDSCKGGVYGFTEEWLAACSGEMAGNDAAGFFSDEHASNLNFGWCASGNEGWE

>Oshox14

MDRYGEKQQQQQMFASYVDASLLAASGEVQGERPRARRRRRRGARCVGGGGGGGEVDGGDPKKRRLSDEQVEMLELSFREERKLETGRKVHLASELGLDPKQVAVWFQNRRARHKSKLLEEEFSKLKHAHDAAILHKCHLENEVLRLKERLVVAEEEVRRLRSAAGSHTASGEGGDIMGLGGSGACVAGSPSSSFSTGTCQPPSFGGGDHLGDDDLVYVPEYGGYADNSVVEWFSLYGLI

>Oshox16

MEEGGRGVKRPFFTTPDELLEEEYYDEQLPEKKRRLTPEQVHLLERSFEEENKLEPERKTELARKLGLQPRQVAVWFQNRRARWKTKQLERDFDRLKASFDALRADHDALLQDNHRLHSQVMSLTEKLQEKETTTEGSAGAAVDVPGLPAAADVKVAVPDAEEPALEEAAAAFEEQQEQQVKAEDRLSTGSGGSAVVDTDAQLVVGCGRQHLAAVDSSVESYFPGGDEYHDCVMGPMDHAAGGIQSEEDDGAGSDEGCSYYADDAGVLFADHGHHHHHQHADDDEEDGQQISCWWMWN

>Oshox20

MRSPAALLPVVADGGGGVGVEEEMDVDEDMAMCGGRGGGGGEKKRRLSVEQVRALERSFETENKLEPERKARLARDLGLQPRQVAVWFQNRRARWKTKQLERDYAALRQSYDALRADHDALRRDKDALLAEIKELKGKLGDEDAAASFSSVKEEEDPAASDADPPATGAPQGSSESDSSAVLNDAEILPHKPAPAAAADAAASEETEAVVTGAALLHHAEVFFHGQLLKVDDDEAAFLGDDGAACGGFFADEHLPSLPWWAEPTEQWTT

>Oshox21

MASNGMASSPSSFFPPNFLLHMAQQQAAPPHDPQEHHHHHHHGHHGHHHEQQQQQQHHHHLGPPPPPPPHPHNPFLPSSAQCPSLQEFRGMAPMLGKRPMSYGDGGGGGDEVNGGGEDELSDDGSQAGEKKRRLNVEQVRTLEKNFELGNKLEPERKMQLARALGLQPRQVAIWFQNRRARWKTKQLEKDYDALKRQLDAVKAENDALLNHNKKLQAEERRSMWVSVLAERLLESVPFSPPRIVALKGREAASELINLNKETEASCSNRSENSSEINLDISRTPPPDAAALDTAPTAHHHHHGGGGGGGGGGGMIPFYTSIARPASGGGVDIDQLLHSSSGGAGGPKMEHHGGGGNVQAASVDTASFGNLLCGVDEPPPFWPWPDHQHFH

>Oshox22

MDRGDHHHLQQQHQFLMPPPAPVVPPQLCMPAMMADEQYMDLGGGGAAAAPGRGGAGERKRRFTEEQIRSLESMFHAHHAKLEPREKAELARELGLQPRQVAIWFQNKRARWRSKQLEHDYAALRSKYDALHSRVESLKQEKLALTVQLHELRERLREREERSGNGGAATTAASSSSCNGSGSEEVDDDDDKRNAAAGCLDLEPPESCVLGGATCATPADVSVESDQCDDQLDYDEGLFPESFCATPELWEPWPLVEWNAVA

>Oshox23

MASNGAAAGAMAPFFPPNFLLQMQQPLPLHHQHLQDHAHGGHGGHHLLPPPPPSLSPFLPDLAMDAPPPPMYEASGGDGGGGGAASEDEEDGCGGGGGGGGGEKKRRLSVEQVRTLERSFESGNKLEPERKAQLARALGLQPRQVAIWFQNRRARWKTKQLEKDFDALRRQLDAARAENDALLSLNSKLHAEIVALKGGAAAAGGGGSSCRQEAASELINLNVKETEASCSNRSENSSEINLDISRPAPPPPPPPANESPVNRGIPFYASIGRGGAGGVDIDQLLLRGGHSPSPAAVTTPPPPKMELGITGNGGGADAAAAGAGSFGGLLCGAVDEQPPFWPWADGHHHFH

>Oshox24

MESDCQFLVAPPQPHMYYDTAAAAVDEAQFLRQMVAAADHHAAAAGRGGGDGDGGGGGGGGGERKRRFTEEQVRSLETTFHARRAKLEPREKAELARELGLQPRQVAIWFQNKRARWRSKQIEHDYAALRAQYDALHARVESLRQEKLALADQVDELRGKLNERQDQSGSCDGGGAEGDDDDKRNSVMNASSSGLVEEDYVSCLAVPVVDVSEDGSAACGGSSYEYDHHLDYLGGGQLPDPFCGMPDLWEIWPMVEWNAVA

>Oshox25

MAASSEVSIDPDQWFYIYSLERVAYVCERVGSPVMEEAELRRRRRKRPFLTTTHDELELQMEDLVDELYGVDEQGSSSAAARKRRLTAEQVRALERSFEEEKRKLEPERKSELARRLGIAPRQVAVWFQNRRARWKTKQLELDFDRLRAAHDELLAGRTALAADNESLRSQVILLTEKLQANGKSPSPSPAPAEQTAVPAAPESAKSFQLEEGRRLYDAAGSTTTTNGGGGGVAMPAARVAAARAASNDSPESYFAGARSPPSSSEDDCGGAGSDDDYPSSSVLLPVDATLVGDAFEHAVAATVAADEEAPLNSWEWFWN

***Zea mays***

>Zmhdz1

MESGRLIFNAPGSGAGQMLFLDCGAGGGPGGGLLHRGGRPMLGLEEGRGVKRPFYTSPDELLEEEYYDEQLPEKKRRLTPEQVHLLERSFEEENKLEPERKTELARKLGLQPRQVAVWFQNRRARWKTKQLERDFDRLKASFDALRADHDALLQDNHRLRSQVVSLTEKLREKEATEGDANGAAAALPAVGVVRASVADDVEEPEPEPEPEAEAAAFEERQVKSEDRLSTGSGGSAVVDAADALLYNGRIGGAAVDSSVESYFPGAEVQDQYHGCGTMGPVTHGAGGIQSDDDDGAGSDEGCSYYADEEAAAAFLSEHTHHADGDDEDAGQVSWWWMWN

>Zmhdz2

MDPSAVSFDSGGTRRGGGAQMLLFGGGGSANSNGFFRGVPMAVLGMDDATRVGKRPFFTTHEELLEEEYYDEQAPEKKRRLTAEQVQLLERSFEEENKLEPERKTELARRLGMAPRQVAVWFQNRRARWKTKQLETDYDRLKAAYDALAADHQGLLADNDNLRAQVISLTEKLQGKETYPSATTAAQEVDQPDEHTAVSGTEELLAQQLKDNLHSSGDCTGHGTLSSEEDDGGVVSDEGCSFALPDAMFAAGFTHHGAEEVQLANWTSMFWN

>Zmhdz3

MHPSAVSFDSGDGRRRGGAQMLLFGGGGSANSNGFFRGVPMAVLAMDDATRVGKRPFFTTHEELLEEEYYDEQAPEKKRRLTAEQVQLLERSFEEENKLEPERKTELARRLGMAPRQVAVWFQNRRARWKTKQLETDYDRLKAAYNALAADHQGLLADNDSLRAQVICLTEKLQGKETSLSATIAAQEVDQPNEHTTGTEKLLAQQLKDDLHSSCGCTGHGALSSEEEDGGVVSDEGCNFDLPDAMFAVGVTNHGAEEAQLANWTSWFWN

>Zmhdz4

MDRPDHQQQQFFMPTTVQVPQPQQQQQQLCVPMLDEPPSSFLAGRGGGGASGRGERKRRFTEEQIRSLESMFHAHHAKLEPREKAELARELGLQPRQVAIWFQNKRARWRSKQLEHDYALLRAKFDDLHAHVESLKQDKLALTTQLSELSERLRERDDRAAAAGGGGRETMASSSSCIGGGGEEEAEDDKRNVLLFGCVDMEPPAESCVLVGSTCAALADVSVESECDDQHLHYDDEFPESYCAMPELWEPWPLVEWNAVA

>Zmhdz5

MRPMASNGMASSLSPFFPPNFLLQMQQTPSDHDPQEHHHLPAPPLHLHHNPFLPSSQCPSLQDFRGMAPMLRKRPTMYCGADDVGVGGEEASCGATNEDEVSDDGSLQAVGPGEKKRRLNVEQVRTLEKNFELGNKLEAERKLQLARALGLQPRQVAIWFQNRRARWKTKQLEKDYDALRRQLDAVKADNDALLSHNKKLQAEILALKGREAAGGSSELINLNKETEASCSNRSEDSSEINLDISRTPPASEGPMDHPPPPPPHHAAGGGLIPFYPSVGGRHSIAAGVGMDQLLHASTPKLEQHGDGCAVAVQAAETAGSFGNLLCGVDETPPFWPWADHHHFH

>Zmhdz6

MERGDCQFTVVPPRQYDEAQFMHQLMVAGDQQDPAGAGRGAAAAGGERKRRFTEEQVRSLETTFHARRAKLEPREKAELARELGLQPRQVAIWFQNKRARWRSKQLEHDYAALRAQFDAMHARVESLRQEKIALAAQVDELRGRLNERQDQSGSCEVNDAEAAADDKRNNSTSSLVQDDGATPPPAAVDASEDSAATGEYYDHVAYEYDGLHDPFVCATPDLWDTWPLLEWNAVA

>Zmhdz7

MDPASSLISSSAVPFPAAGDGGGQRQVFLFGGGFVGGAPPLVGGAADGRRKRPFQLTAHDELQLQLQLQLDDELCGLDYELHGPQQERTTKRRLTAEQVRELELSFEEEKRKLEPKRKLEPERKSELARRLGIAPRQVAVWFQNRRARWRSKQLEQDFDRLRAAHDDLIAGRDALLADNDRLRSQVITLTEKLQTKESAAAAAEPGEQTVAAQATAYAVLEGDKLCSGSAAAPAAAGTNDSPESYFAGARSPPSSPEDDDRAFFFPPDALLNNWAWVWNDRQY

>Zmhdz8

MKRPRGASPPLAAMTNPNDDGYGVVGMEADADADADEEMMACGGGGEKKRRLSSEQVRALERSFEVENKLEPERKARLARDLGLQPRQVAVWFQNRRARWKTKQLERDYSALRQSYDALRHDHDALRRDKDALLAEIKELKAKLGDEEAAASFTSVKAEPAASDGPPPVGVGSSESDSSAVLNDADPPVAEAPVPEVRGTLLDAPGAVAANHGGVFFHGSFLKVEEDETGLLDDDEPCGGFFSVEQPPPMAWWTEPTEHWN

>Zmhdz9

MEGDDDGPEWMMEVGGAGATGKGKGGALDKNKKRFSEEQIKSLESMFATQTKLEPRQKLQLARELGLQPRQVAIWFQNKRARWKSKQLERDYSALRDDYDALLCSYESLKKEKHTLLKQLEKLAEMLHEPRGKYSGNADAAGAGDDVRSGVGGMKDEFADAGAAPYSSEGGGGGKFAHFTDDDVGALFRPSSPQPSAAGFTSSGPPEHQPFQFHSGCWPSSTEQTCSSSQWWEFESLSE

>Zmhdz10

MSWRECVRNEDLSPCACVFPDDGYGVLGMEADVDVDVDEEMMAFGGGGGGEKKRRLSAEQVRALERSFEVENKLEPERKARLARDLGLQPRQVAVWFQNRRARWKTKQLERDYAALRRSYDALRLDHDALRRDKDALLAEIRELKAKLGDDEDAAASFTSVKAEPAASDGPAPAGVGSSEISDSSAVLNDADPPVAEAPAPAPEVQGTLLVAPAGPVAGAAAANHGGVFFHGSFLKVEEDETGLLDDDGPCGGFFAVEQPPAMAWWNEPAEHWN

>Zmhdz11

MRPMASNGMASAPSPFFPPNFLLQMQQTPSDHEHQEQHHHHHHHKHHLAAPPLHPHHNPFLPSSQCPSLQDFRGMAPIMLGKRPMYGADVQVVGGEEVNGCGGVNEDELSDDGSQAGGEKKRRLNVEQVRTLEKNFELGNKLEPERKLQLARALGLQPRQVAIWFQNRRARWKTKQLEKDYDALKRQLDAVKADNDALLSHNKKLQAEILSLKGREAGGSSELINLNKETEASCSNRSENSSEINLDISRAPASEAPLDPTPPPGAGGGGMIPFYPPSVGGRPASAAGVDIDQLLHTSAPKLEQHGSGGAVVVQAAETASFGNLLCGVDEPPPFWPWADHQHFH

>Zmhdz12

MDGAEDDGTEWMMHGAGGKGKGGGALDKNKKRFSDEQTKSLESMFATQAKLEPRQKLQLARELGLQPRQVAIWFQNKRARWKSKQLEREYSALRDDYHALLCSYESLKDEKRALLKQLEKLAEMLHEPPQGKYYGGNADDVRSGGVGGTKEEEESTDACAGAALYSSECAGGGRFIAHFLADDDVGAALFRPPSSPQPTAGLLTSSGPPEHQPFQFHSGCCWPSSSAEQTCSGSQWWELESLSE

>Zmhdz13

MKPMATNGMAPSFYPANFLLQMQQALPHHYQQQEQHHEGHDDEDHEAAHHLLAPPPAALVSPFLHDFGGAMAAAPPPPMLAGGFGGKRMYPDVGGMAGDDSHHLHAEPQQQQEQQASDDEEEGSAAVGGGERKRRLSVDQVRTLERSFEVANKLEPERKAQLARALGLQPRQVAIWFQNRRARWKTKQLEKDYDALRRQLDAARAENDALLSHNKKLQAEIMALKGGGGGGGRQEAASELINLNVRETEASCSENNSSEINGLDVSRPDPAAGESPAMNSYRGGLPFYASAAARADIHHQLLHSGGQPSPAAAAPPKMELAPGSPPATAGGGGGTFGSLLCGAAVDEPPPFWSWADGHHTCFQ

>Zmhdz14

MKRQSKRPTASRESPETGEKQKLAFAEEEAPPARKVEPDDEAEMDEDEDELGPGLGGGRAARSPCGLGEKKRRLALEQVRALERCFETDNKLDPDRKARIARDLALQPRQVAVWFQNRRARWKTKTLERDFAALRARHDALRADCDALRRDKDALAAEIRELRQKLLPKPEATVKLEATTGNDTAEERRQATAGAPPAGACKDGSSDSDSSVVFSDVEASPYSGGAAFEPPALAGLGAPFLDTSVAPTGCSSPPVFVETKWQQHGPTQYPFPFDSYKACAGYGFTEEWLASSDLIGSDGGAAGFFSEDHASSLNFNWCPSGIQGWE

>Zmhdz15

MGCEEEERLLFPSFVFPESFAEAATPGSGGEQKKARQRRRRKPRPAEGGEGADEQARKRRLSDDQARFLELSFRKERKLETPRKVQLAAELGLDAKQVAVWFQNRRARHKSKLMEEEFSKLRAAHDAVVLHNCHLETELLKMKDRLAEVEEEKTKLVAAAAAAAGGAAGAGSSSPSSSSFSTVTHHPAAALQVGQFGVEPEEAADLAYMTEYAYNSYMNMMDLAPAYFGGVVYDYDHFN

>Zmhdz16

MKRPTSRGSSMVSGQATTDLQEDSCRSMEPHGVDGDATAAAERDDVDAGAYDEEVDEEEELAGSRGGLGEKKRRLAADQVRALERSFEVDNKLDPERKARIARDLSLHPRQVAVWFQNRRARWKTKQIERDFAALRVRHDALRVECDALRRDKDALAAEIKELRGMVEKQMEVKLESAAEELLPVATRSAAAAAVYNKDGSTDSDSSAVFNEEASPYPYSGAALDHQQQQETSHPLGFTGFTSFLTSSFPSVYHGDSHLDQEADGFFSAAAAGDGFFAEEQSAGIGSWYGGEGW

>Zmhdz17

MAAWVSCLHLWPLSLSLSLSLSTPSLTPVLQSCSARKEIGITANSFVRATRLLLFQDPCRSLTIESPPFPNPFQSSAAKCRSQMKRQSKRPTASRDSPETGTVDKQLALAESLTARKTEPDELGEKKRRLAPDQVRALERCFEADNRLDPDRKARVARDLALNPRQVAVWFQNRRARWKAKALHRDFAALRARHDALRRDKDALAAEIRELRQKLAEPAAVKTEATGSDTAEARPTTTTTAGAPAPPAGGCKDGSSDSDSSVVLNDDVDASPYSGAAFEQPGAFAGFGAPFLAASAAAAPPPTGCSSPPVFETKWQHGPTTYAFESYKSCCAGYGFTEEWLASSDVVGSDGAAGFFSEDHASSLNFSWCASGAQDWE

***Sesamum indicum***

>SiHDZ07

MSGGIKKSSRNINRRRFSDDQIKSLETMFETESRPELRLKQHLANKLGLQPRQVAIWFQNKRARSKSKQIEQEYSMLKSNYDKLASQFEALRKENQNLLTQVDRLRRLADNGDGDEHDNKNHFTLSSSENPRLLLDINCQELCIPTCSDLNRKVGYLEEEADALNMAQIAGGSLASPENRGSFDSSTFLDNTGSGSQWWNF

>SiHDZ09

MSTEMAFFHPNFMLQTPHQDEHTHPSTSLPPLLPSCSTPQEFHGVAPLLGKRSSMGFSTGIDVCEEMNNHGEDELSDDGSQVGEKKRRLNMEQVKTLEKNFELGNKLEPERKMQLARALGLQPRQIAIWFQNRRARWKTKQLEKDYDLLKRQFEAVKAENDALQAQNQKLHAEIMALKNKEPTESINLNKETEGSCSNRSENSSEIKLDISRTPAIDSPLPAHPTNAGRPLFPSSLIRPSGVAQQLFHNPSRPEIQCPKMDQSVKEESLCNMFCGMDDQTGFWPWLEQQHFN

>SiHDZ10

MEGCKSYDDSHMENVLLKNERIPCTSEVLDCLWVPNSSPPFHVSGSAAVVNFEEIRGENSTERPFFTQVDSKVENLSEEFDGCFHQPEKKRRLTVEQVQFLEKSFEVENKLEPDRKVQLAKELGLQPRQVAIWFQNRRARYKTKLLEKEYGSLKASFDKLKADFDTLSAENEKLKNEVQLLTQKMLLRDNGKPKLESFDPISPLDLQPENPAFSPQAAACKQEDGASSAKSDILDSDSPRHTDGNFPAILDPVGSSHDFEPDPSDFSQEENESLSRSLLQPTCFPKLEVECYDDLQPNSCNLGFPVQDQGTWFWQY

>SiHDZ15

MTCEMAFFHSNFMLQTPPHQEDHDQNHPSTSLPPISLPSCTPQEFHGGVASLLAGKRSSMSFSTGIDVCEDHMNNHNHGEDELSDDGSQVGEKKRRLNMEQVKTLEKNFELGNKLEPERKMQLARALGLQPRQIAIWFQNRRARWKTKQLEKDYDLLKRQFEAVKAENDALQAHNHKLHAEILALKSREPTESINLNKETEGSCSNRSENSSEIKLDISTTTPAATCTESPLSTHPIPRALFPSTSSLSRPGGVVAHQLFHNSSSRPEIHHCPKMDQTVKEESLCNMFVGMDDQTGFWPWLEQQNFN

>SiHDZ16

MESPHEENPHRSTRKTSKNARRFTNEQIRSLETIFEMETKLEPRKKHEVARDLGLQPRQVAIWFQNRRARWKSKQLEQEYKVLKTNYDGLCMQFDHLKREKQSLQMQISKSRAFPQLQELTDLVKNLDNVGISDGDAELKEDPNSSNEGMDHNDAMYTDDEETVTKYFKETEEQEPLSWGTEQVEISHLESPETWCNDLSSGSLFDQSSCKSNWWESLKI

>SiHDZ24

MDSGRIFFDSSSNGNLLFLGNGDSIFRGGSRVMNMGESSKRRPFFGTAEDLYDEEYYDEQLPEKKRRLTPDQVNLLEKSFEEENKLEPERKTELAKKLGLQPRQVAIWFQNRRARWKTKQLERDYDHLKSSYDSLVSNYDSIIKENNKLKAELLFLTEKLQPKESTQEEGFDIQNPNPTPAPYLQLIMKVEDRLSTGRQRRGQLLDSGDSYLEYQQGCMASVEGVKSEEDEGSEEGGSYFSDVFAAPEPQEPVGWFMWP

>SiHDZ27

MEVYDDSRISNALLKNERLPCTSEVLDSLWVQNSSPSFHASASMVNFANVQGETKTERPFLPQIDTKAENCNEGALDGCSFHHPEKKRRLTPNQVQFLEKSFDEENKLEPERKLQLAKELGLQPRQVAIWFQNRRARYKTKLLEKEFDSLKSSYDRLKADYDTLYKENEKLKTEVNSLAEKSRLRDNGKPKTEQCDDPISPLDLQPNKPICSQNAAQTVASKQEDAASSAKSDVFDSDSPHYTDGNHSAALEPADSSHVFEPDASDFSQDEDDSFNRSCLMPSLSFQNWKLNALMIYSRTRAIWDSLFRIKGHGSGSIELRGSGLDVGGIFGSCMLMLLC

>SiHDZ31

MMSDWMENSCVGNLNTMAKKKKIKNKRRFSDEQIRSLEVMFESESKLEPKKKAQLANELGLQPRQVAIWFQNKRARWKSKHMEKEYAILLANYNNLASQVEALKEQNQSLLIKLQKLKNEMMGKSEGQIQEIARKGITDDDGESDHTKIQLSEVTNVGTRSLEEILSDDDSSIRPVLFGLDEENELLKLVEPAAADSSLTSAEDWGSLDSDDLINQPTDHDCYQWWDFWS

>SiHDZ35

MDWNANLRVPNFVSRPDNSLSFLYNYNYNDQFPAGLEIKQPTMQSSTQHTMLPTFMEKSNSSMNIYGQDPKKKRLSTEQLESLENSFQEEIKLDPDRKMKLARELGLQPRQIAVWFQNRRARWKGKQLERLYDALKQEFDAVSREKHKLQQEVLALRAILKDQVAKKQVSTGYTEVISGEETVESTSIPSSDNTPAPPENVPHHQIPECNYVFNVEDYNQVMMTPTPYNWPPLPSCP

>SiHDZ37

MDEFRCPQTRKQQSKHRKRLSQDQVRLLETNFNFNNKLDPDRKSRLALELGIPPRQVAIWYQNKRAREKNQSLEADHKTLQQQLENVVAENARLEGEVERLKAELNKIHDVLNAFNSSTCCEEVGSSSLSSEQNLEKELYAALIGVRDPFVAPDGYDFFG

>SiHDZ39

MMINPEFDDQMVLISGQYYPVGIISQFVQEQGAAVEESKPRRRRRKKNGGGGDGDGEAVMMRKRKLSEEQANMLEKSFGSEHKLESERKERLAAELGLDPRQVAVWFQNRRARWRSKKLEEEFSKLRSHHDSTLIEKSRLETELVKVKEQLQEAEKEIQRLSERWDHGFSSNSPSSSTNMEQPPLLGFEGLENVFCVPHDNSYGHGFEWDNFYYL

>SiHDZ40

MKRLSSSDSLGALMSICPNSTDEHNNPMYTREFQTMMEGLDEEGCVEESGHVSDKKRRLSVDQVKALEKNFEVENKLEPERKVKLAQELGLQPRQVAVWFQNRRARWKTKQLERDYGVLKANYDGLKLNFEKLQHDNEALLKEIGELKSKLNQDGNSESKVSDAEVKSKHETQLKENGTEISFETLNGDSNGGIPTRGVSVFGDFKDGSSDSDSSAILNEDNNNSPHQFLDIQNGSNSISFQFCSESKPGETTTAIAGLGEAHKNYHTQFVKIEEHNFFGEESCTSFFTDDQAPTLPWYCSDDWN

>SiHDZ41

MAFVPAESMFRNHEDSHFPSSSPCAPQDFHGVPEFLMRRSMSFSGMERPEDQLQPEDEMSDDGSQLLGEKKRRLNLEQVKALEKSFELGNKLEPERKMQLARALGLQPRQIAIWFQNRRARWKTKQLEKDYDMLKRQFEALKADNDALKSQNKKLQTQLLALKTRDSTGSGAINLNKENEGSWSNVSENSCEVNLNTTSTDSTLYPHPHHHQTNKNPVINFPSAIGPSSLTQLLQRPDHHLQLCQKMDHAPVSNDGFGNMFGAGIEENQDFWPWPEQQNFN

>SiHDZ43

MKPMNPEEVDDQMVLISQYYPDGIYSQFVPEQGAAMNSKPRRRRKKNKGDRGGAGDGVMRKRKLSEEQVNMLERSFGSEHKLESERADRLAAELGLDPRQVAVWFQNRRARWKSKKLEEEYSKLKSEHETAVVEKCRLETELLKLKEQLQEAEKEIHRLSERWDGVSSNSPSSSFSIEAMDPPFLGEFGMEGLGNVFYVPEAEENYAQGLEWNTFYYM

>SiHDZ44

MKRSLGSSDSLGALMSICPNSTDENSPKGDYPMYTREFQTMLEGLDEDCGGGCVEETGQVSEKKRRLRVDQVKALEKNFEVENKLEPERKVKLAQELGLQPRQVAVWFQNRRARWKTKQLERDYGVLKANYDALKHKYEQLQRDNDSLLNEILELKSKLNEGDGEHDSKVSVKEEPLVSETEAKTEIEAPFIELNFENCRNELTTMMREASVFVDFKDGSSDSSDSSAILNEDNNNSPVGAAISSSAGGVLHHSPPAHDDEFLDNVHGIGLSSSAISLHQLSDSKVYQPPQFVKIEEHNFFGEECCSSLFSDDQPPTLHWDDCSDVWN

>SiHDZ45

MDWNGNLRVPFVSRPAAVENSYNFHYNFNYDQFPVLEMKQPPMLTTPQQAMLPTLADKNNSMMHNFGQDPKKKRLSNEQLESLENSFQEEIKLDPDRKMKLAKELGLQPRQIAVWFQNRRARWKAKQLERLYDALKREYDAVSRDKQKLQQEVMALRAILKEHLAKKQVLSTGYTDISGEETVESTSIPTSNDPSPPTTTNSLHQINADHQCNNNYGLNVDDYNNPVMMPPYWAAAAAPSCP
