# Supplementary material for: Analysis of the HD-Zip I transcription factor family in Salvia miltiorrhiza and functional research of SmHD-Zip12 in tanshinone synthesis
Source: PeerJ. 2023 Jun 27;11:e15510. doi: 10.7717/peerj.15510 (PMC10312201; doi:10.7717/peerj.15510)
Supplement: Table S4 [file peerj-11-15510-s005.docx]

**Table S4.** The motif sequence information of SmHD-Zip I

| Motif | E-value | Site | Width | logo |
| --- | --- | --- | --- | --- |
| 1 | 1.1e-737 | 24 | 50 | 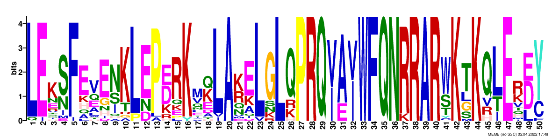 |
| 2 | 1.1e-136 | 25 | 21 | 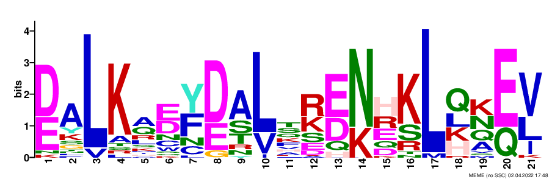 |
| 3 | 8.7e-099 | 20 | 20 | 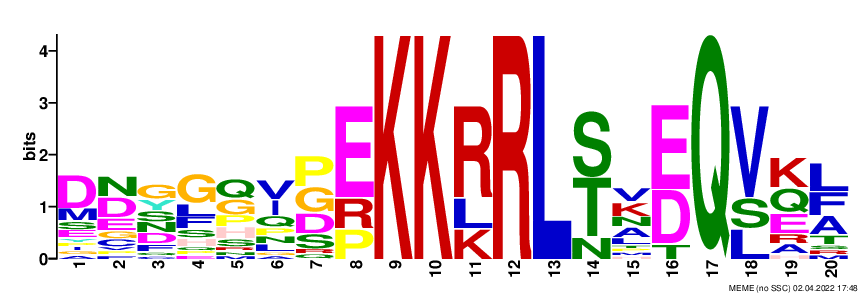 |
| 4 | 2.9e-036 | 4 | 49 | 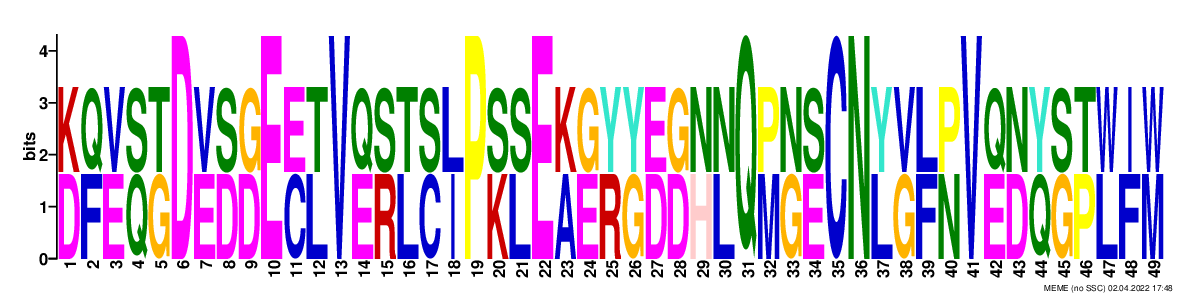 |
| 5 | 2.9e-029 | 3 | 29 | 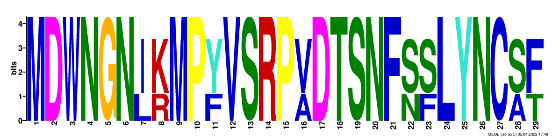 |
| 6 | 2.5e-016 | 5 | 14 | 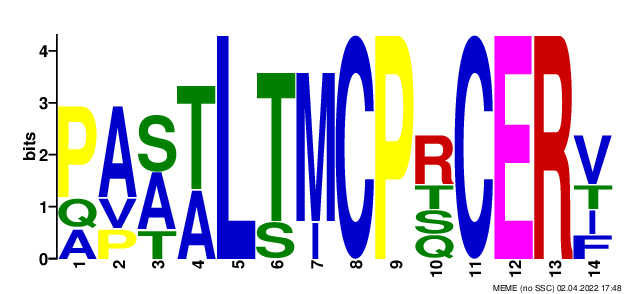 |
| 7 | 2.1e-014 | 5 | 11 | 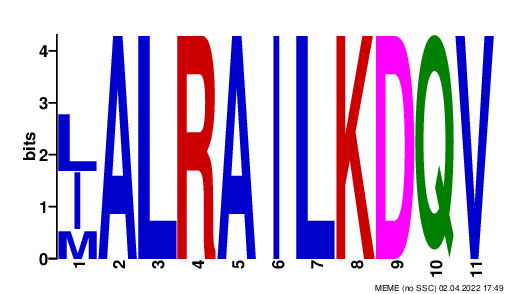 |
| 8 | 3.4e-013 | 7 | 15 | 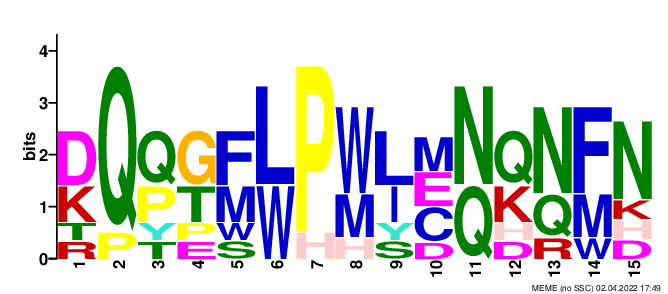 |
| 9 | 2.9e-008 | 3 | 28 | 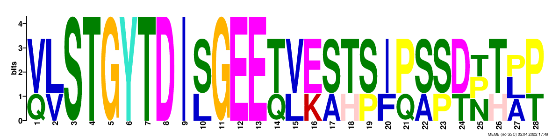 |
| 10 | 7.5e-007 | 2 | 25 | 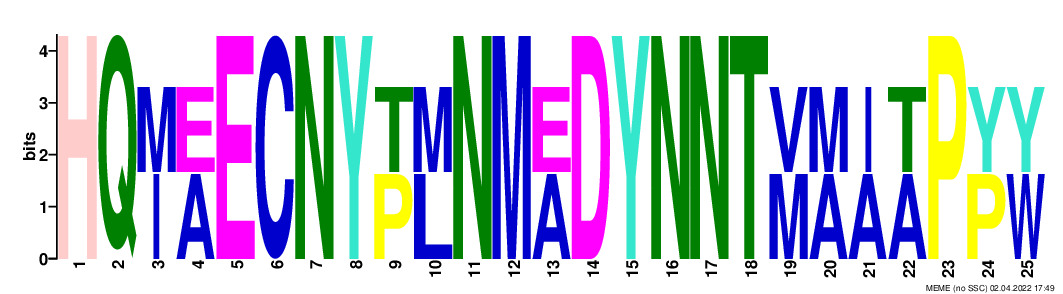 |
| 11 | 1.7e-007 | 3 | 23 | 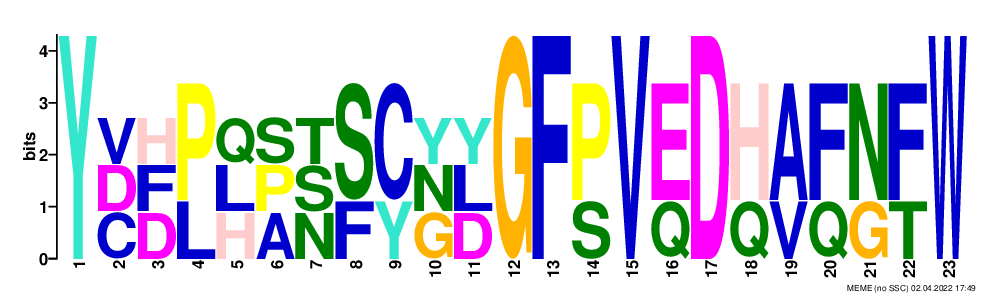 |
| 12 | 1.3e-005 | 2 | 14 | 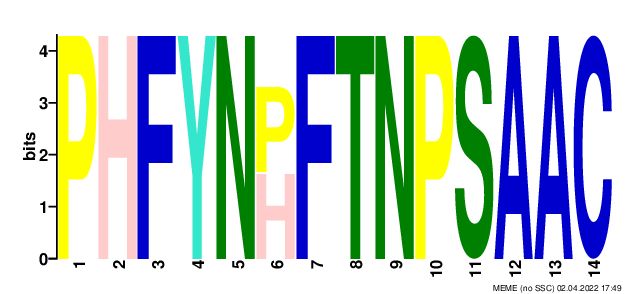 |
| 13 | 5.9e-004 | 2 | 25 | 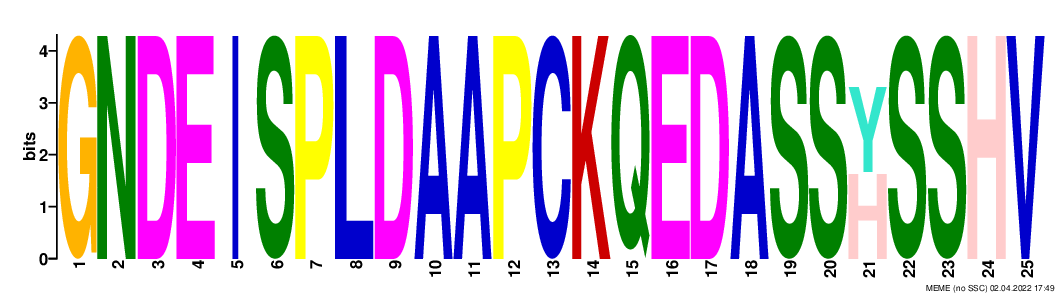 |
| 14 | 4.6e-003 | 3 | 12 | 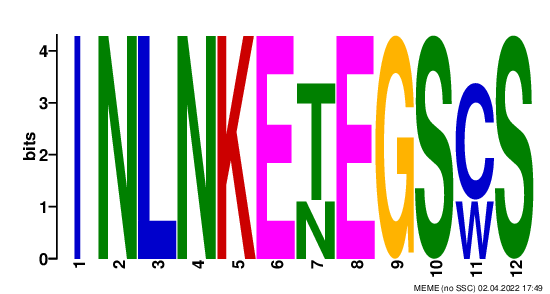 |
| 15 | 2.8e-002 | 2 | 11 | 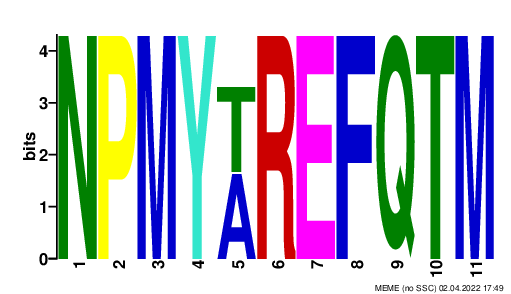 |
| 16 | 2.9e-002 | 3 | 15 | 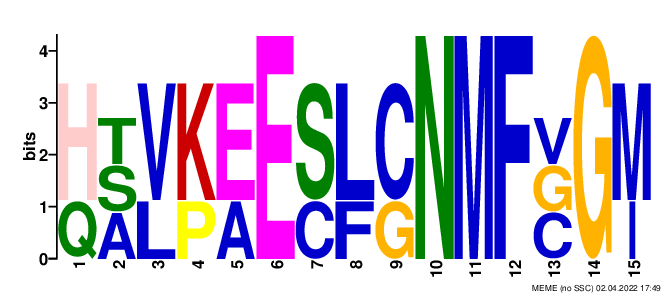 |
| 17 | 4.5e-002 | 2 | 21 | 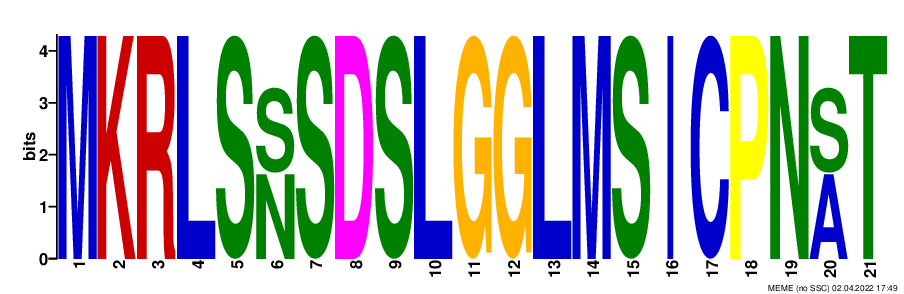 |
| 18 | 6.0e-002 | 2 | 21 | 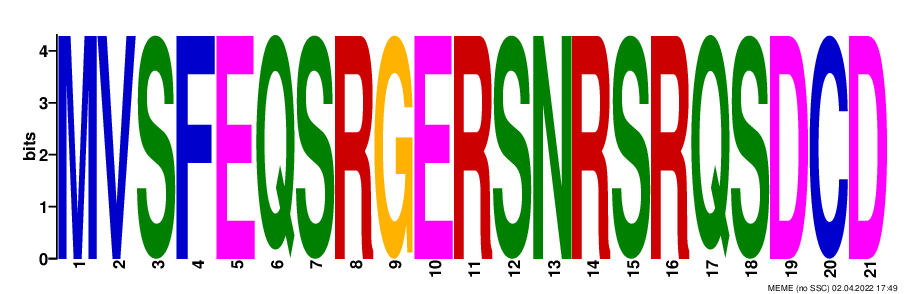 |
| 19 | 2.9e-001 | 2 | 11 | 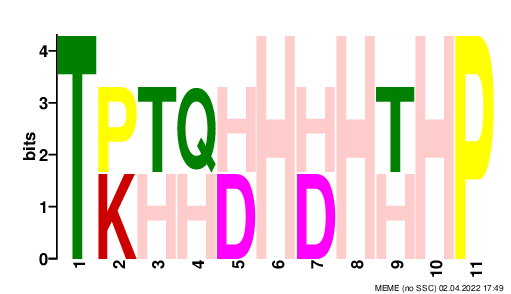 |
| 20 | 2.9e+000 | 2 | 10 | 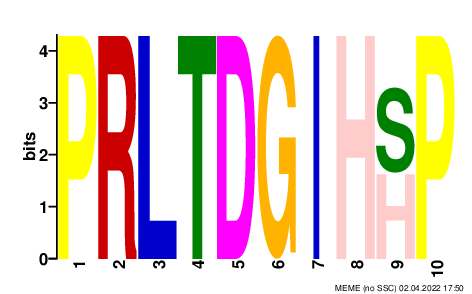 |
