# Supplementary material for: Analysis of the HD-Zip I transcription factor family in Salvia miltiorrhiza and functional research of SmHD-Zip12 in tanshinone synthesis
Source: PeerJ. 2023 Jun 27;11:e15510. doi: 10.7717/peerj.15510 (PMC10312201; doi:10.7717/peerj.15510)
Supplement: Figure S1 — Hairy roots were cultured in a 6,7-V liquid medium for 18 days before being treated. The 2−ΔΔCT method was used to be an evaluation of the relative expression. SmActin was used as the internal standard. ** and * indicate significant differences compared to the control (0 h) at p < 0.01 and textitp < 0.05, respectively. [file peerj-11-15510-s006.docx]

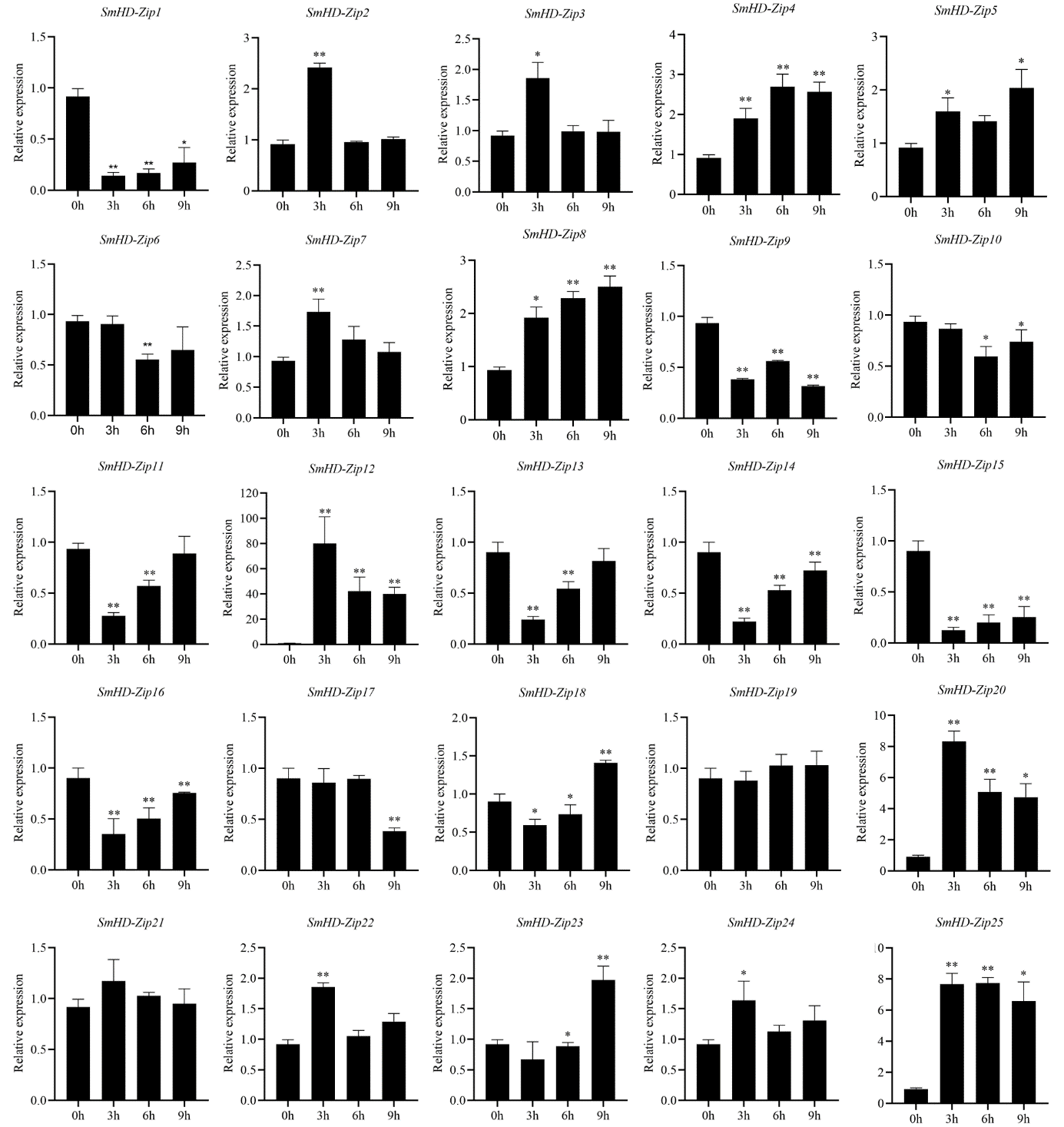

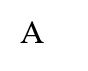

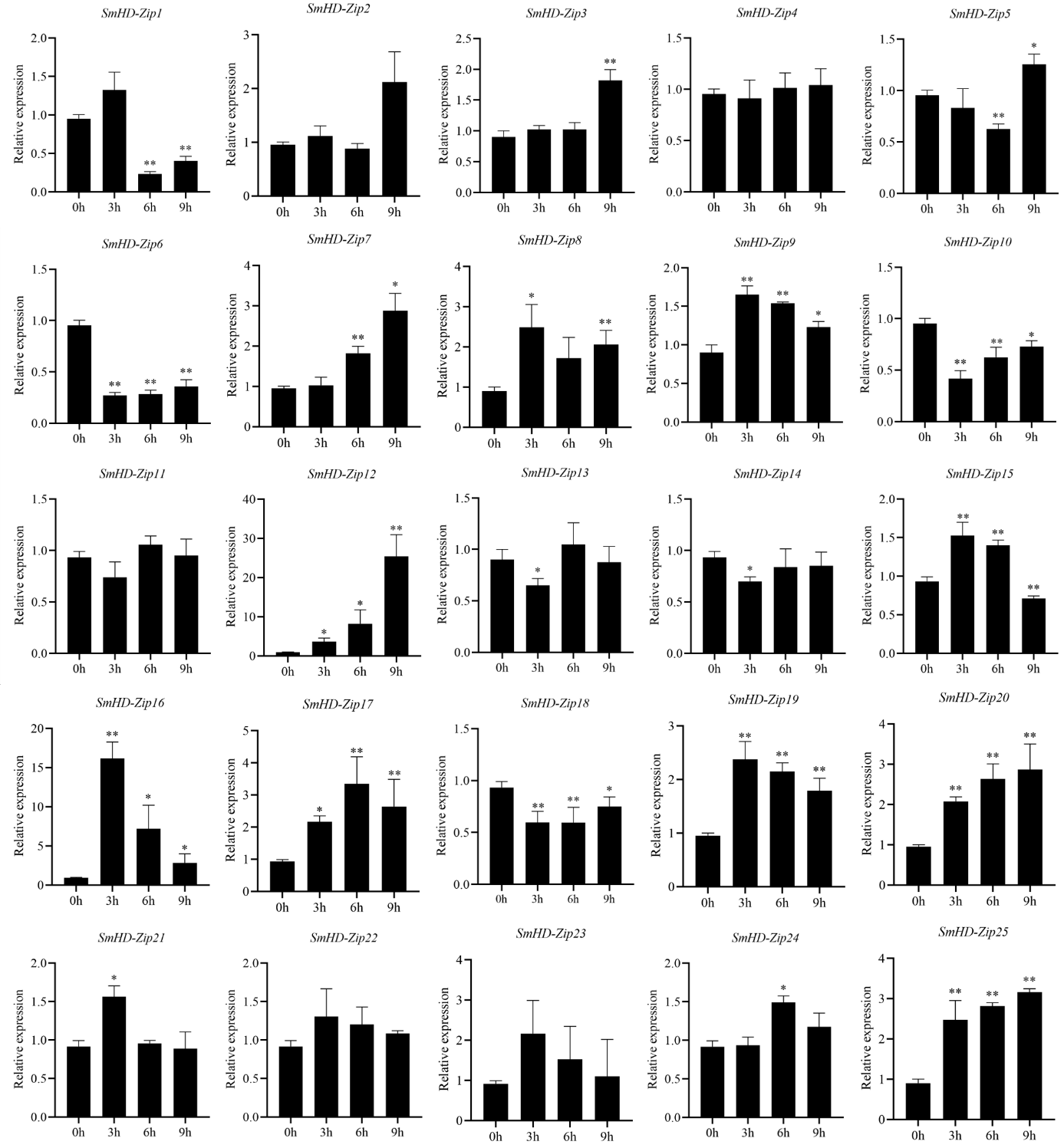

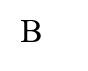

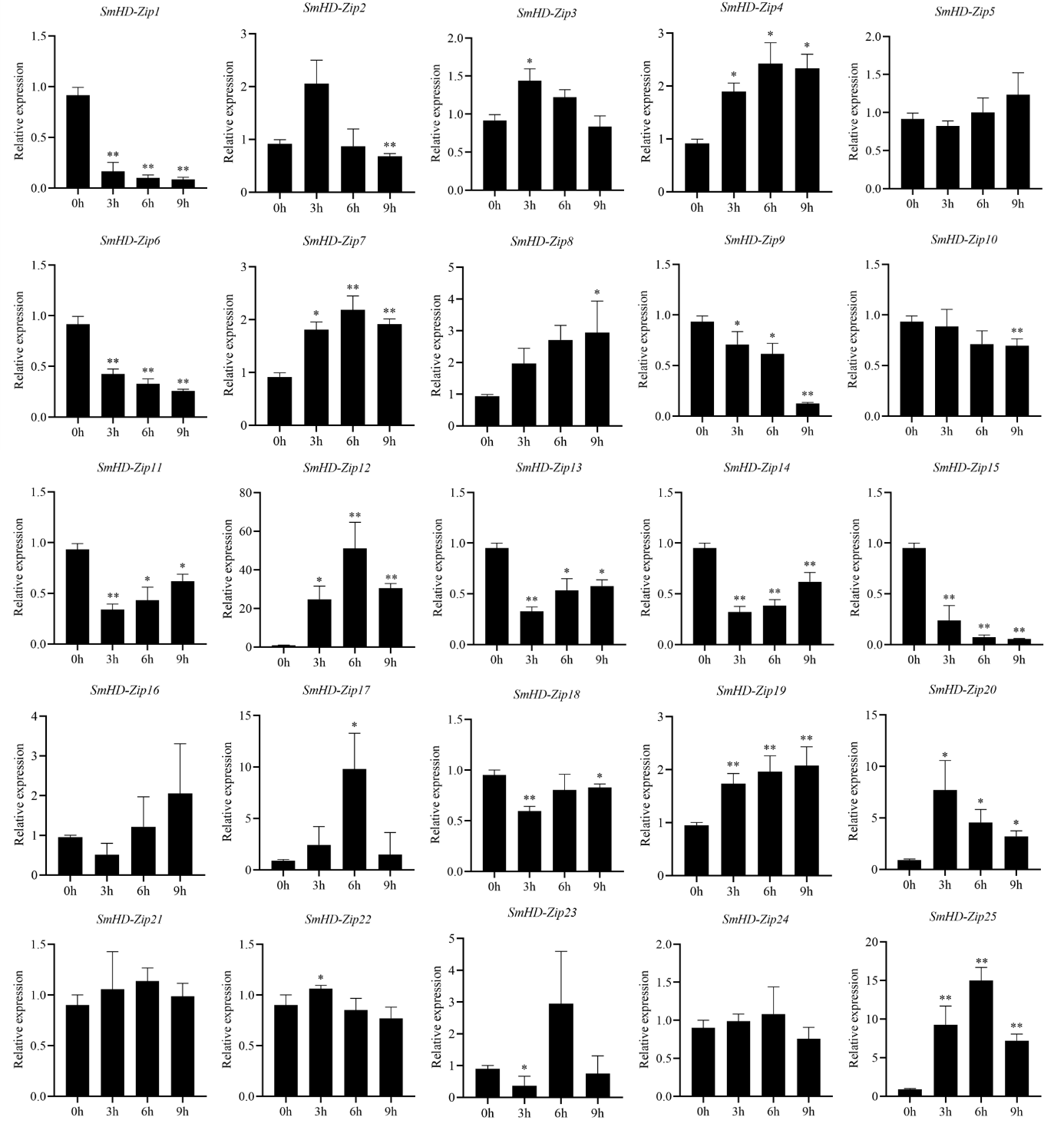

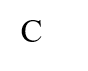


**Figure S1.** The expression patterns of the *SmHD-Zip I* at 0, 3, 6, and 9 h after treated with ABA (A), PEG (B), and NaCl (C). Hairy roots were cultured in a 6,7-V liquid medium for 18 days before being treated. The 2^−∆∆CT^ method was used to be an evaluation of the relative expression. *SmActin* was used as the internal standard. ** and * indicate significant differences compared to the control (0 h) at *p* < 0.01 and *p* < 0.05, respectively.
