# Supplementary material for: Analysis of the HD-Zip I transcription factor family in Salvia miltiorrhiza and functional research of SmHD-Zip12 in tanshinone synthesis
Source: PeerJ. 2023 Jun 27;11:e15510. doi: 10.7717/peerj.15510 (PMC10312201; doi:10.7717/peerj.15510)
Supplement: Figure S2 — M, DL-2000; 1, WT; 2, EV; 3-10, Overexpression of SmHD-Zip 12. (B)PCR analyses of transgenic hairy root lines by SmHD-Zip12-specific primers. M, DL-2000; N1: H2O; N2: WT-PC: Recombinant plasmid; 1–17: Overexpression of SmHD-Zip 12. (C)Relative expression analysis of SmHD-Zip 12 genes by qRT-PCR in in transgenic lines and WT. ** and * indicate significant differences compared with the wild type (WT) at p < 0.01 and p < 0.05, respectively. [file peerj-11-15510-s007.docx]

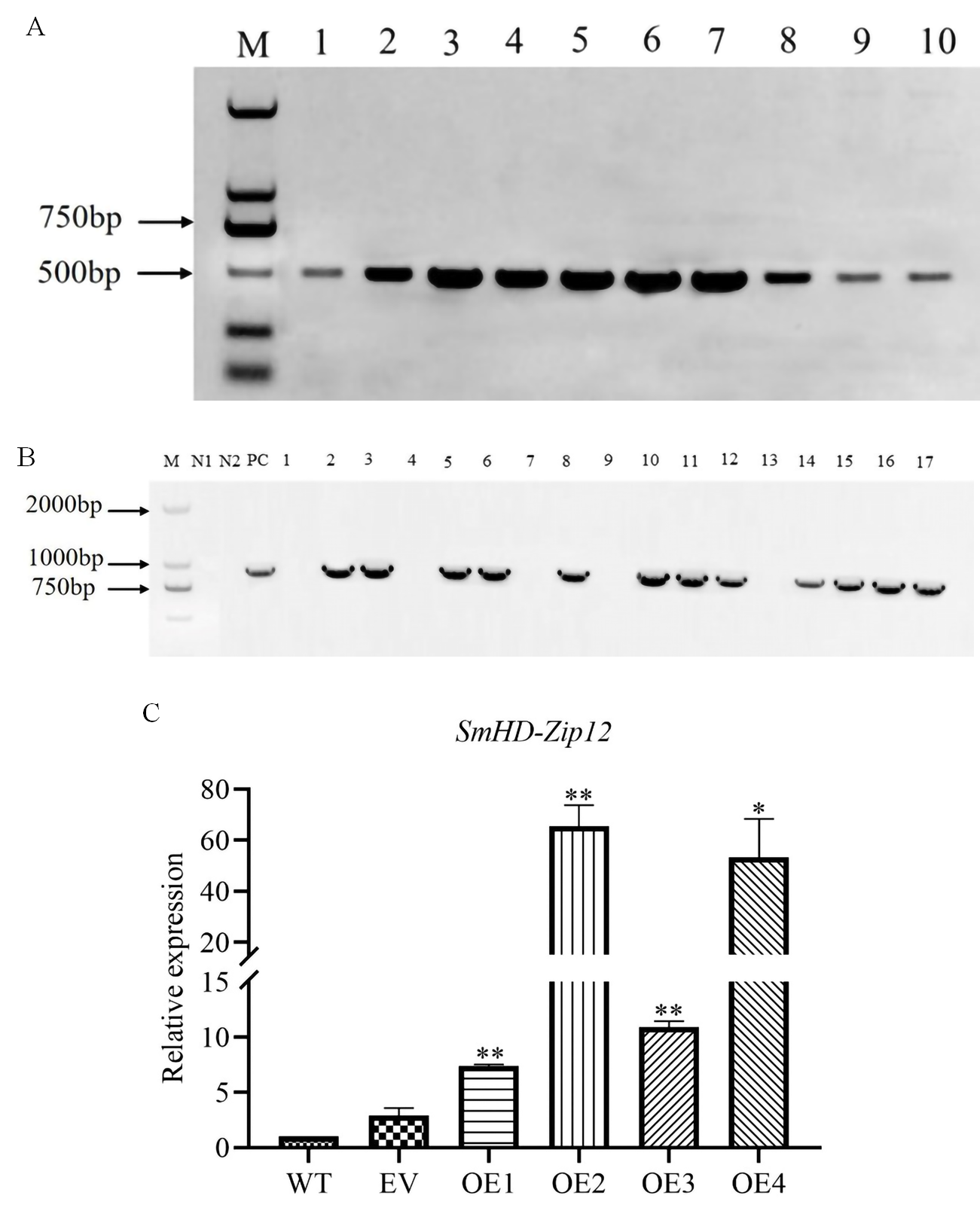

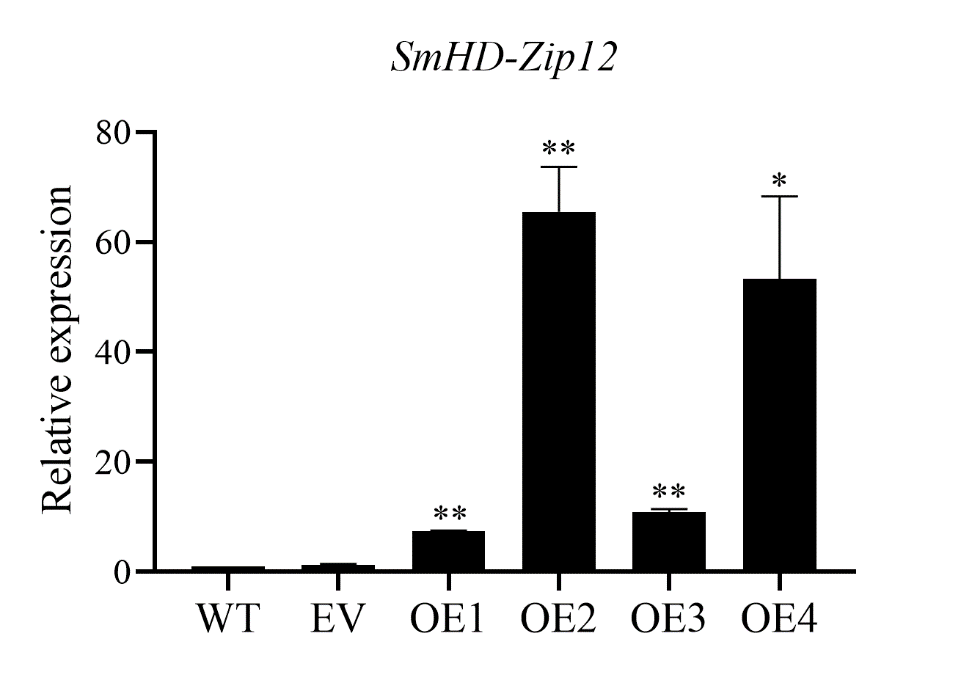

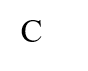


**Figure S2.** Identification of *SmHD-Zip12* overexpression hairy roots. (A)Identification of hairy roots by rolB. M, DL-2000; 1, WT; 2, EV; 3-10, Overexpression of *SmHD-Zip 12.*(B)PCR analyses of transgenic hairy root lines. M, DL-2000; N1: H_2_O; N2: WT；PC: Recombinant plasmid; 1-17: Overexpression of *SmHD-Zip 12*.(C)Relative expression analysis of *SmHD-Zip 12* genes by qRT-PCR in in transgenic lines and WT of *S. miltiorrhiza* hairy roots. ** and * indicate significant differences compared with the wild type (WT) at p < 0.01 and p < 0.05, respectively.
